# MR1 displays the microbial metabolome driving selective MR1-restricted T cell receptor usage

**DOI:** 10.1126/sciimmunol.aao2556

**Published:** 2018-07-13

**Authors:** Melanie J. Harriff, Curtis McMurtrey, Cara A. Froyd, Haihong Jin, Meghan Cansler, Megan Null, Aneta Worley, Erin W. Meermeier, Gwendolyn Swarbrick, Aaron Nilsen, Deborah A. Lewinsohn, William Hildebrand, Erin J. Adams, David M. Lewinsohn

**Affiliations:** 1 VA Portland Health Care System, Research and Development, 3710 Southwest U.S. Veterans Hospital Road, Portland, OR 97239, USA.; 2 Department of Pulmonary and Critical Care Medicine, Oregon Health & Science University, 3181 Southwest Sam Jackson Park Road, Portland, OR 97239, USA.; 3 Department of Microbiology and Immunology, University of Oklahoma Health Sciences Center, Oklahoma City, OK 73104, USA.; 4 Department of Biochemistry and Molecular Biology, University of Chicago, Chicago, IL 60637, USA.; 5 Oregon Health & Science University Medicinal Chemistry Core, Portland, OR 97239, USA.; 6 Department of Pediatrics, Oregon Health & Science University, Portland, OR 97239, USA.; 7 Department of Molecular Microbiology and Immunology, Oregon Health & Science University, Portland, OR 97239, USA.

## Abstract

MR1-restricted T cells (MR1Ts) are a T cell subset that recognize and mediate host defense to a broad array of microbial pathogens, including respiratory pathogens (e.g., *Mycobacterium tuberculosis*, *Streptococcus pyogenes*, *and Francisella tularensis*) and enteric pathogens (e.g., *Escherichia coli* and *Salmonella* species). Mucosal-associated invariant T (MAIT) cells, a subset of MR1Ts, were historically defined by the use of a semi-invariant T cell receptor (TCR) and recognition of small molecules derived from the riboflavin biosynthesis pathway presented on MR1. We used mass spectrometry to identify the repertoire of ligands presented by MR1 from the microbes *E. coli* and *Mycobacterium smegmatis*. We found that the MR1 ligandome is unexpectedly broad, revealing functionally distinct ligands derived from *E. coli* and *M. smegmatis*. The identification, synthesis, and functional analysis of mycobacterial ligands reveal that MR1T ligands can be distinguished by MR1Ts with diverse TCR usage. These data demonstrate that MR1 can serve as an immune sensor of the microbial ligandome.

## INTRODUCTION

The T cell receptor (TCR), a heterodimer composed of αβ or γδ chains derived by recombination in the thymus, enables T cells to recognize non-self ligands presented in the context of conventional and nonconventional major histocompatibility complex (MHC) molecules. Conventional MHC molecules have high amino acid diversity concentrated in their peptide binding grooves, which allows the presentation of a broad array of peptides. In contrast, the nonconventional CD1 molecules are monomorphic and present lipid and glycolipid antigens (1). Like CD1, MR1 is a nonconventional, monomorphic MHC molecule, with its gene located on human chromosome 1 (2). Unlike CD1, MR1 presents small-molecule metabolites from microbes such as *Mycobacterium tuberculosis*, *Salmonella typhimurium*, and *Candida albicans* (3, 4). MR1-restricted T cells (MR1Ts) can be defined by their dependence on ligands displayed by MR1 (3–7). Mucosal-associated invariant T (MAIT) cells, a subset of human MR1Ts, were defined by the use of a semi-invariant TCR consisting of a single α chain rearrangement (TRAV1–2 and TRAJ33) paired with a limited number of β chains (TRBV6 and TRBV20) (7, 8).

Restriction to a monomorphic antigen presentation molecule along with limited TCR diversity suggested that MAIT cells were limited to recognizing a small repertoire of ligands and were unable to discriminate between ligands (9). Furthermore, the semi-invariant nature of the MAIT cell TCR has been used to argue that MAIT cells lack properties of immunologic memory, specifically antigen-driven clonal expansions reflective of antigenic exposure and persistence. That the known activating ligands for MAIT cells are derived from 5-amino-6-d-ribitylaminouracil (5-A-RU), a biosynthetic intermediate in the riboflavin biosynthesis pathway (6, 10), and that only microbes that synthesize riboflavin were initially demonstrated to stimulate MAIT cells were viewed as further proof of a limited ligand repertoire. However, examination of the MR1 binding pocket shows that the known ligands do not fill the entire binding pocket (11), suggesting that other ligands that are more structurally diverse may also be presented by MR1. Moreover, identification of an MR1T clone that recognizes *Streptococcus pyogenes*, which lacks the riboflavin biosynthetic pathway (12), suggests a broader MR1 ligand repertoire than only riboflavin pathway-based molecules. In addition, recent evidence of more diverse TCR usage among MR1Ts and the ability of some MR1T clones to recognize the MR1 ligand 6,7-dimethyl-8-d-ribityllumazine (RL-6,7-diMe) better than other clones raises the possibility that MR1Ts can discriminate between MR1-activating ligands and specific microbes (12–14).

Previous work identifying MR1-activating ligands has used a directed approach, reacting small molecules from the glycolysis pathway with 5-A-RU to generate neoantigens (10). However, this approach does not enable broad sampling of microbial metabolites. To explore the broader MR1 microbial ligandome, we developed a strategy that would sample the microbial ligandome for MR1 binders and then performed functional and biochemical analysis of these ligands. We chose to focus on two divergent microbes, *Escherichia coli* and *Mycobacterium smegmatis*, to broadly sample the potential repertoire of MR1 ligands. We used liquid chromatography–mass spectrometry (LC-MS) and molecular networking to identify new MR1 ligands and demonstrated that some ligands are inhibitory, whereas others are activating. In support of MR1Ts having antigen selectivity, we show that discrete TCRs can discriminate between microbial ligands. Moreover, our molecular networking analysis indicates broader diversity within the MR1 microbial ligand repertoire, including ligands that are not derived from 5-A-RU.

## RESULTS

### hpMR1 tetramers loaded with a heterogeneous mixture of microbially derived ligands can be used to both identify MR1Ts and activate them ex vivo

To define the microbial MR1 ligandome, we established a new strategy where recombinant MR1 is expressed in insect cells cocultured with two distinct microbes recognized by MR1Ts, *E. coli* and *M. smegmatis*. We developed a chimeric version of soluble MR1 [human platform MR1 (hpMR1)] composed of the human α1 and α2 platform domain with bovine α3 and β2m domains (fig. S1A). The hpMR1 protein is biochemically stable, is highly expressed (fig. S1B), and recapitulates the ligand binding and TCR interactions of human MR1 (fig. S1C). Insect cells expressing hpMR1 were left without bacteria or cocultured with live *E. coli* or *M. smegmatis*. hpMR1 expressed in the absence of any bacteria (hpMR1^−bac^), or following coculture with either *E. coli* (hpMR1^+EC^) or *M. smegmatis* (hpMR1^+MS^), had distinctly different colors (fig. S1D), suggesting differences in the ligand repertoire.

**Fig. 1.**
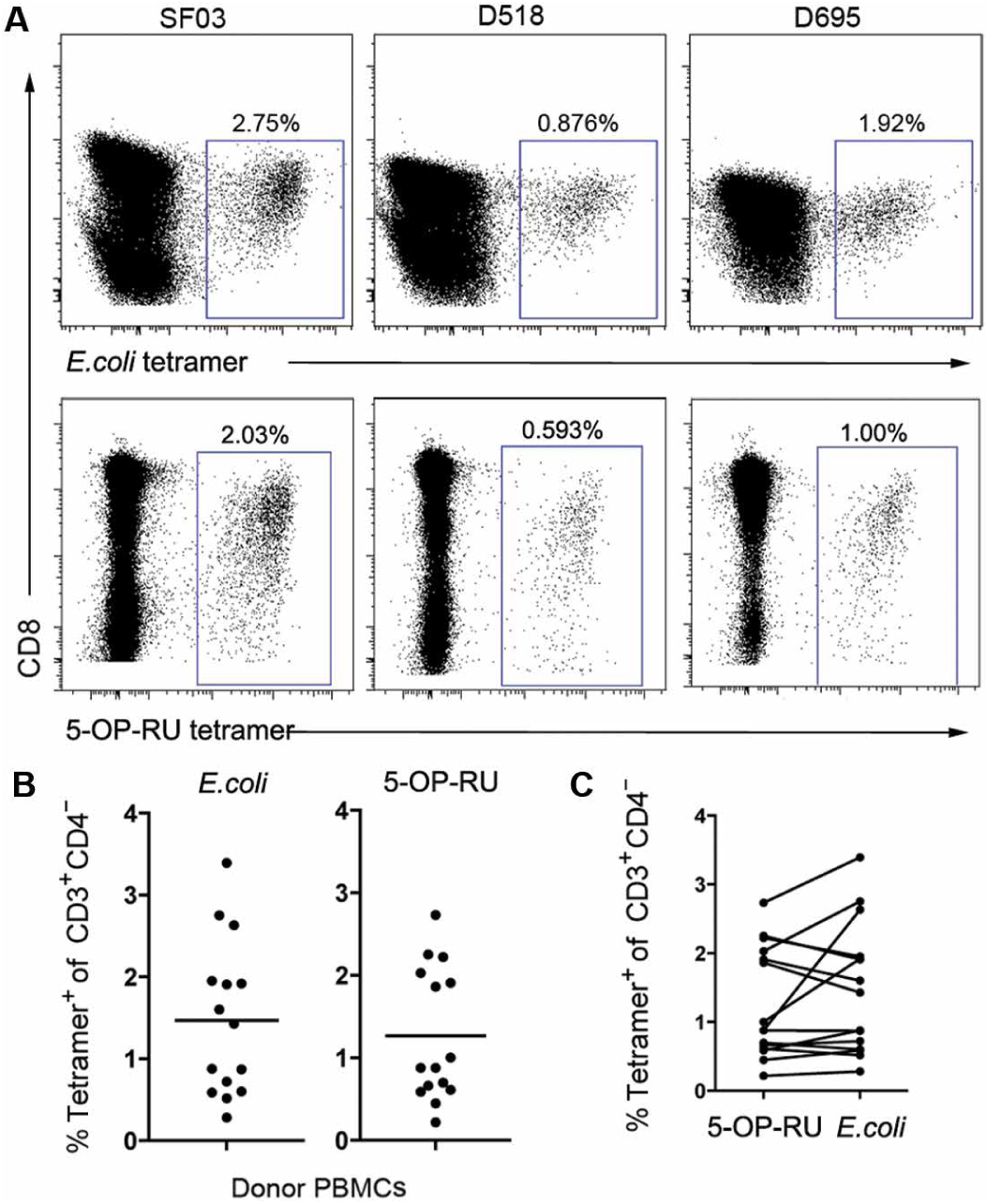
MR1Ts are recognized by hpMR1 tetramers loaded with a heterogeneous mixture of microbially derived ligands. (**A** to **C**) PBMCs from 15 donors were stained with the hpMR1^+EC^ tetramer (12.5 nM per test) or the MR1/5-OP-RU tetramer (1:500; NIH tetramer core) and a panel of phenotypic markers. (A) Representative dot plots for three donors. Blue boxes denote the frequency of CD3^+^CD4^−^ tetramer^+^ cells. All plots are log scales. (B) The graphs depict the frequency of CD3^+^CD4^−^MR1/5-OP-RU or hpMR1^+EC^ tetramer^+^ cells for all 15 PBMC donors. (C) The graphs in (B) are paired to demonstrate the relationship between the hpMR1^+EC^ and MR1/5-OP-RU for each donor.

To analyze whether hpMR1^−bac^, hpMR1^+EC^, and hpMR1^+MS^ were loaded with ligands capable of binding MR1Ts, we generated a tetramerized form of hpMR1. In contrast to the existing MR1 tetramer loaded with a single ligand, 5-(2-oxopropylideneamino)-6-d-ribityluracil (MR1/5-OP-RU) (10), our approach produces tetramers composed of hpMR1 monomers loaded with a heterogeneous population of medium-derived, host cell–derived, and bacterially derived ligands generated in the context of infection. Using an MR1T clone, we first demonstrated that hpMR1 tetramers could specifically stain this clone (fig. S2, A and B). The hpMR1^+EC^ and hpMR1/5-OP-RU tetramers both delineated a distinct population of cells in peripheral blood mono-nuclear cells (PBMCs) of each of 15 donors tested (Fig. 1, A to C, and fig. S2C). For a subset of donors, there was a greater frequency of hpMR1^+EC^ tetramer^+^ cells (average of 15 donors: 1.47 ± 0.94%) than MR1/5-OP-RU tetramer^+^ cells (average of 15 donors: 1.26 ± 0.81%) (Fig. 1C).

**Fig. 2.**
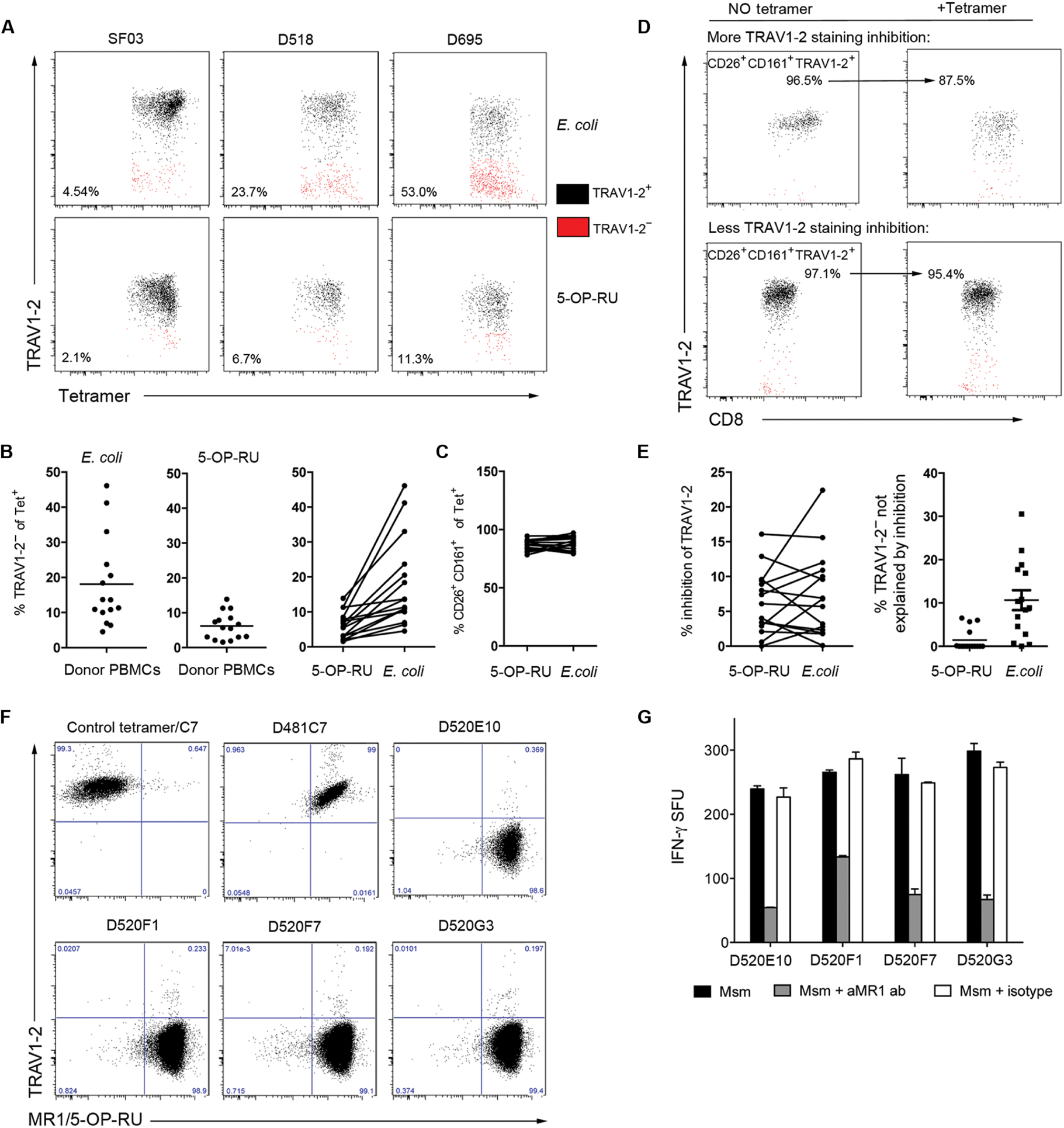
MR1Ts recognized by the hpMR1^+EC^ tetramer are more likely to be TRAV1–2^−^. PBMCs from 15 donors were stained as described in Fig. 1, and the population of tetramer^+^ cells was analyzed for phenotypic MAIT cell markers. (**A**) TRAV1–2 staining of tetramer^+^ cells for three representative donors. Black indicates TRAV1–2^+^ cells, and red indicates TRAV1–2^−^ cells, with the gate defined by the population of CD3^+^CD4^−^TRAV1–2^+^CD161^+^ cells, as shown in fig. S2B. (**B**) The graphs depict the frequency of CD3^+^CD4^−^ tetramer^+^TRAV1–2^−^ cells for all 15 PBMC donors. Right: Frequencies of TRAV1–2^−^ tetramer^+^ cells in left graphs have been paired for each donor. (**C**) Frequency of CD26^+^CD161^+^ cells among tetramer^+^ cells between the tetramers. (**D** and **E**) PBMCs from each donor were simultaneously stained in panels with or without the tetramer to analyze tetramer inhibition of TRAV1–2 staining. (D) Frequency of CD26^+^CD161^+^TRAV1–2^+^ cells for two representative donors. (E) Left: Tetramer inhibition of TRAV1–2 staining is calculated for all donors using the method in (D). Right: For each donor, the inhibition of TRAV1–2 staining by tetramer was used to calculate what proportion of the TRAV1–2^−^ events depicted in (B) are not explained by tetramer inhibition of TRAV1–2 staining. (**F**) TRAV1–2^−^ MR1T clones from D520 (E10, F1, F7, and G3) and a TRAV1–2^+^ MR1T clone (D481C7) were analyzed by flow cytometry for tetramer and TRAV1–2 staining. (**G**) MR1-dependent activation of TRAV1–2^−^ MR1T clones by human DCs infected with *M. smegmatis* (Msm) was measured by IFN-γ ELISPOT assay using the anti-MR1 blocking antibody (aMR1 ab). (F and G) Results are representative of three independent experiments. Error bars represent means and SD from technical replicates.

In our previous studies with the MR1/5-OP-RU tetramer, we showed that 1 to 4% of cells stained by the MR1/5-OP-RU tetramer are TRAV1–2^−^ (12). Here, using a similar analysis, we demonstrate that hpMR1^+EC^ tetramer^+^ cells are in both the TRAV1–2^+^ and TRAV1–2^−^ populations, with a higher percentage of TRAV1–2^−^ cells (hpMR1^+EC^ mean, 18.1 ± 12.76%; range, 4.54 to 46.1%) than observed for MR1/5-OP-RU tetramer^+^ cells (MR1/5-OP-RU mean, 6.2 ± 3.85%; range, 1.58 to 13.9%) (Fig. 2, A and B). Despite this difference in TRAV1–2 staining, there was no difference in CD26 and CD161 staining between the two populations of tetramer^+^ cells (Fig. 2C). To address the concern that we overestimated the frequency of TRAV1–2^−^ cells, we took several approaches. First, to determine whether the tetramer interfered with TRAV1–2 staining, we compared the frequency of TRAV1–2^+^CD26^+^CD161^+^ for all donors in the presence or absence of either tetramer (Fig. 2, D and E). Although there were some instances in which the frequency of TRAV1–2^+^CD26^+^CD161^+^ was reduced in the staining panel containing the tetramer (e.g., Fig. 2D, top), it was not enough to explain the frequency of TRAV1–2^−^ cells (Fig. 2E). Second, we generated several TRAV1–2^−^ T cell clones. Here, a line was generated by expanding TRAV1–2^−^ cells on *M. smegmatis*–infected cells. The line was then sorted for CFSE (carboxyfluorescein succinimidyl ester)-diluted, tetramer^+^ cells, which were then used in a limiting dilution analysis assay to generate T cell clones. We confirmed that these clones were TRAV1–2^−^ yet could be stained with the MR1/5-OP-RU tetramer (Fig. 2F). These clones were then tested for their ability to recognize bacterially infected cells by IFN-γ enzyme-linked immunospot (ELISPOT) assay. As demonstrated in Fig. 2G, all clones produced IFN-γ, and the response was blocked with the anti-MR1 26.5 antibody. Together, these data support the hypothesis that the population of MR1Ts, as defined by bacterially loaded MR1 tetramers, can be distinguished from conventional MAIT cells.

**Fig. 3.**
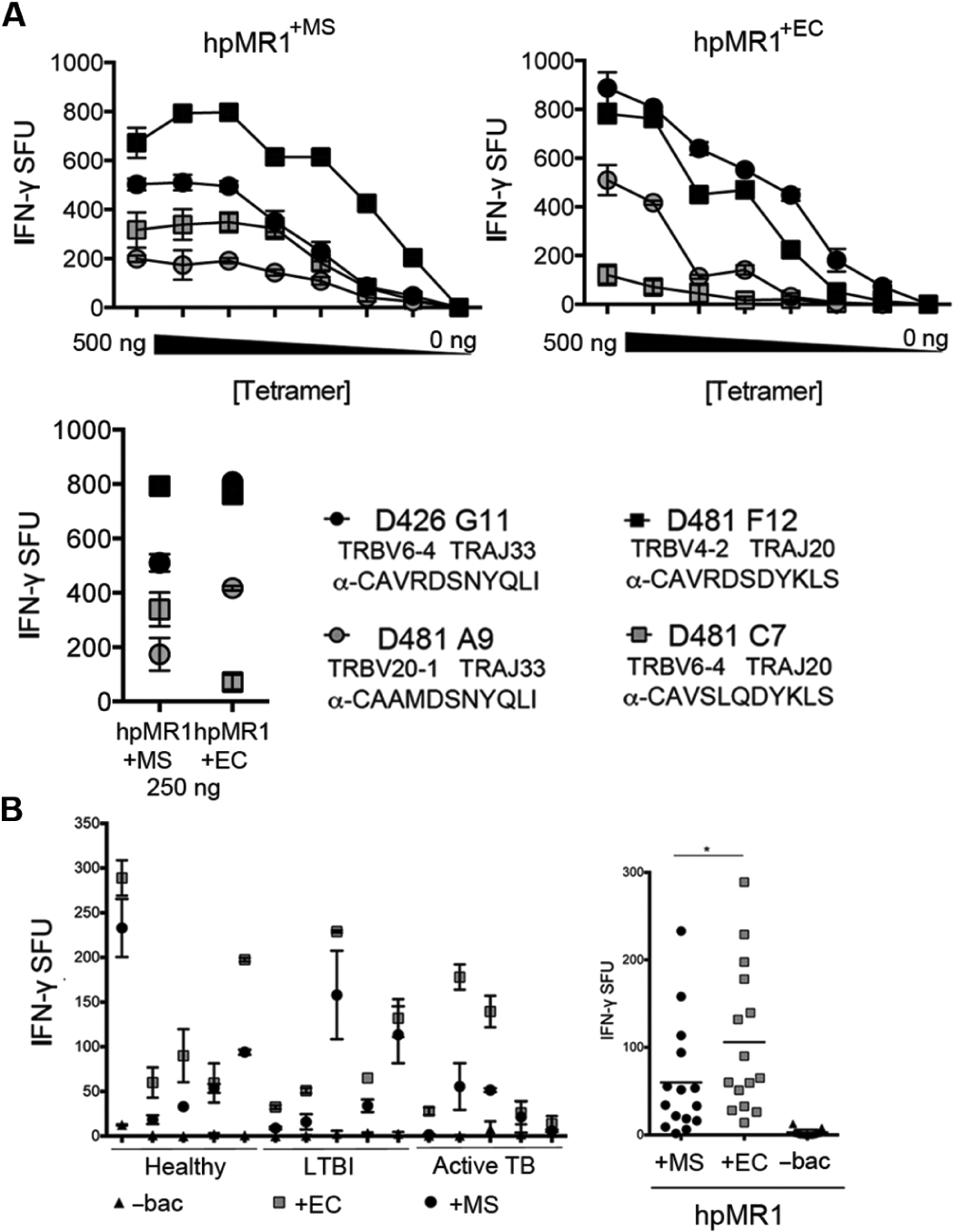
MR1Ts respond to microbially derived ligands loaded on hpMR1 tetramers. (**A**) MR1T clone IFN-γ responses to hpMR1^+EC^ or hpMR1^+MS^ tetramers at 7.8 to 500 ng per well. TRBV, TRAJ, and CDR3 sequences are indicated for each MR1T clone. Results are representative of four independent experiments. Error bars represent means and SD from technical replicates. (**B**) IFN-γ responses from 5 × 10^5^ PBMCs from 15 donors to hpMR1^−bac^, hpMR1^+EC^, or hpMR1^+MS^ tetramers, plotted by individual donor (left) or pooled (right). *P < 0.0001 (paired two-tailed *t* test).

To determine whether hpMR1^+EC^ or hpMR1^+MS^ contained MR1T-activating ligands, we used plate-bound hpMR1 tetramers to stimulate MR1T clones in a modified IFN-γ ELISPOT assay (tetraSPOT), based on an approach similar to that previously used for CD1 (15, 16). Using this assay, we demonstrated that both TRAV1–2^+^ and TRAV1–2^−^ clones responded to MR1/5-OP-RU tetramers with widely varying functional affinities (fig. S3). hpMR1^+EC^ and hpMR1^+MS^ tetramers also robustly stimulated IFN-γ production by a panel of MR1T clones expressing TCRα chains of diverse *TRAV* and *TRAJ* rearrangements (Fig. 3A) (14). hpMR1^−bac^ tetramers did not stimulate MR1T clone responses at any concentration. Having established the presence of MR1T-activating ligands associated with hpMR1, we examined the reactivity of MR1Ts in whole PBMCs using the tetraSPOT assay and found that MR1T responses were observed in each of the 15 donors tested (Fig. 3B). Although the hpMR1^+EC^ and hpMR1^+MS^ tetramers elicited comparable responses among the MR1T clones (Fig. 3A), activation in PBMCs was higher in response to hpMR1^+EC^ [mean, 106 ± 81 IFN-γ spot-forming units (SFU); range, 14 to 289 IFN-γ SFU] compared with hpMR1^+MS^ (mean, 60 ± 63 IFN-γ SFU; range, 6 to 123 IFN-γ SFU) for every donor tested (Fig. 3B).

### Ligands eluted from hpMR1^+EC^ and hpMR1^+MS^ contain both shared and unique ions

The differential activation of the MR1T clones and PBMCs by the hpMR1^+EC^ and hpMR1^+MS^ tetramers led us to postulate that there were unique antigens in hpMR1^+EC^ and hpMR1^+MS^ preparations. We first evaluated the presence and relative abundance of known MR1T ligands. Here, we eluted and identified three of the known activating MR1T ligands [5-OP-RU, reduced 6-hydroxymethyl-8-d-ribityllumazine (rRL-6-CH_2_OH), and 7-hydroxy-6-methyl-8-d-ribityllumazine (RL-6-Me-7-OH)] in hpMR1^+EC^ and hpMR1^+MS^, but not hpMR1^−bac^, by MS/MS (fig. S4, A to H), although we could not distinguish rRL-6-CH_2_OH and 5-OP-RU because they have identical chemical formulas and fragment spectra (6, 10, 11). The 5-(2-oxoethylideneamino)-6-d-ribitylaminouracil (5-OE-RU) and RL-6,7-diMe ligands were not found in any of the hpMR1 preparations. Using extracted ion chromatogram area underneath the curve (AUC) analysis, we found that rRL-6-CH_2_OH/5-OP-RU and RL-6-Me-7-OH were 3.0- and 2.8-fold lower, respectively, in hpMR1^+MS^ as compared with hpMR1^+EC^ (fig. S4, I and J). These data demonstrate that despite similar activation of MR1T clones, there are relative quantitative differences between *E. coli* and *M. smegmatis* in the loading of canonical MR1T bacterial ligands.

**Fig. 4.**
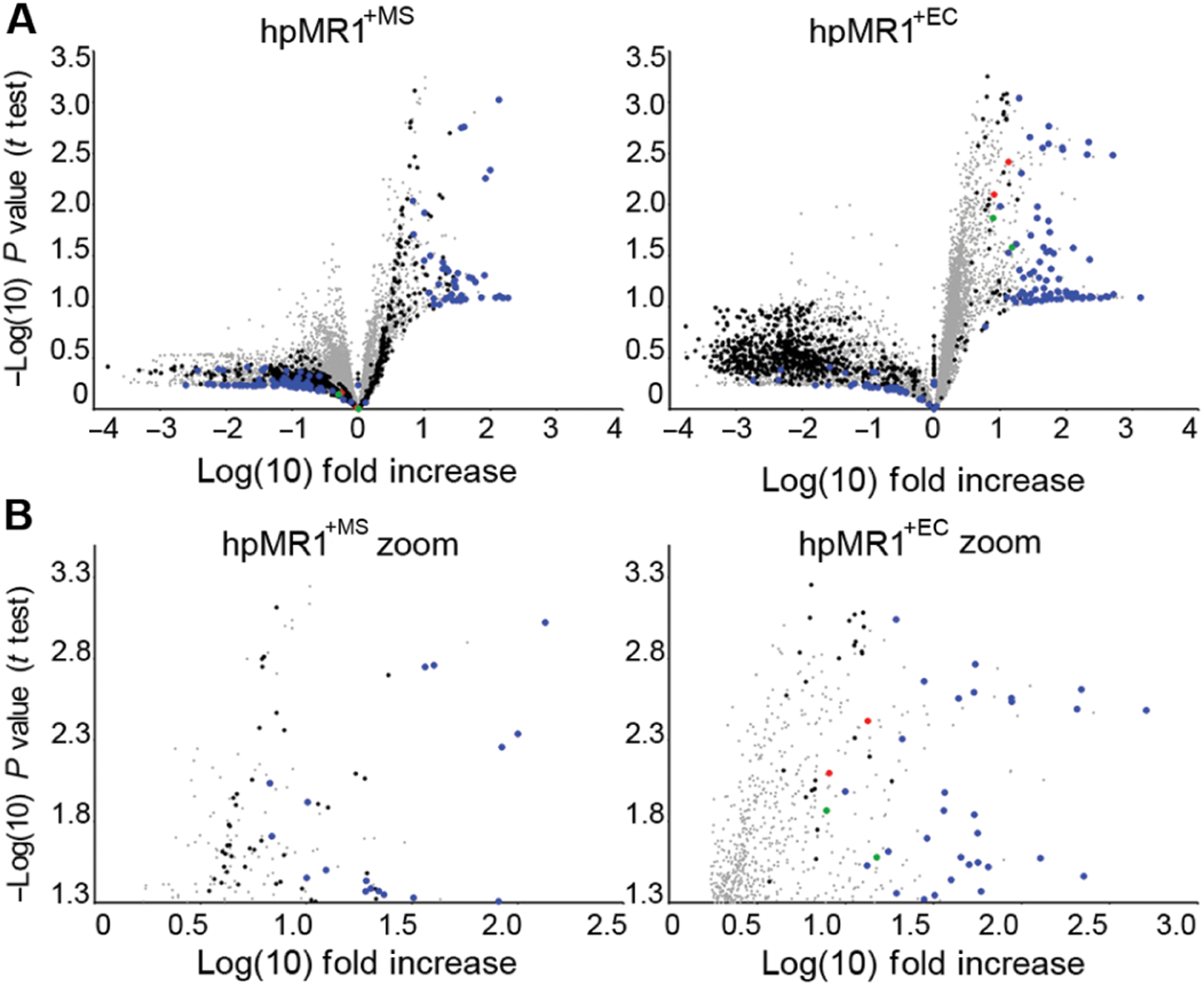
Ligands eluted from hpMR1^+EC^ and hpMR1^+MS^ contain both shared and unique ions. Intensities from all observed MS1 ions in triplicate injections of hpMR1^+MS^, hpMR1^+EC^, hpMR1^−bac^, and T22 were determined using extracted ion AUC analysis. For all ions, average intensities in hpMR1^+MS^ (left) and hpMR1^+EC^ (right) ions were compared with the combined average intensity of all other samples and are plotted as the log(10) of the fold increase. *P* values were obtained with a *t* test and plotted as the inverse log(10). (**A**) Plot of all ions for hpMR1^+MS^ (left) and hpMR1^+EC^ (right). (**B**) Only significantly (*P* ≤ 0.05, −log *P* = 1.3) increased ions for either hpMR1^+MS^ (left) or hpMR1^+EC^ (right). Red and green dots, previously identified MR1T ligands; blue dots, hpMR1^+EC^ or hpMR1^+MS^ unique ligands; black dots, hpMR1 ligands; gray dots, all other ligands. Results are representative of three independent experiments.

To further explore the MR1 ligandome, we compared all ligands eluted from hpMR1^−bac^, hpMR1^+EC^, and hpMR1^+MS^. Fold increase was calculated for each ion/ligand in either hpMR1^+MS^ or hpMR1^+EC^ compared with all other samples and plotted on a volcano plot (Fig. 4A). To control for background ions, we collected data on the non-MR1 class I MHC molecule T22, which does not present antigen (17, 18). In negative ion polarity, a total of 970 ions were observed above the T22 background intensity in hpMR1^−bac^, hpMR1^+EC^, or hpMR1^+MS^ preparations and were considered putative MR1 ligands (black dots; table S1). Of these, 127 ions were observed at >10-fold increased intensity in hpMR1^+EC^ or hpMR1^+MS^ compared with hpMR1^−bac^ and were considered putative microbial-derived ligands (blue dots). Twelve bacteria-derived monoisotopic ions were unique to hpMR1^+MS^, and 29 bacteria-derived monoisotopic ions were unique to hpMR1^+EC^ (Fig. 4B and table S1). These data demonstrate that disparate bacterial species produce distinct ligands for MR1.

### Global Natural Products Social Molecular Networking assists in the identification of novel hpMR1 eluted ions

**Fig. 5.**
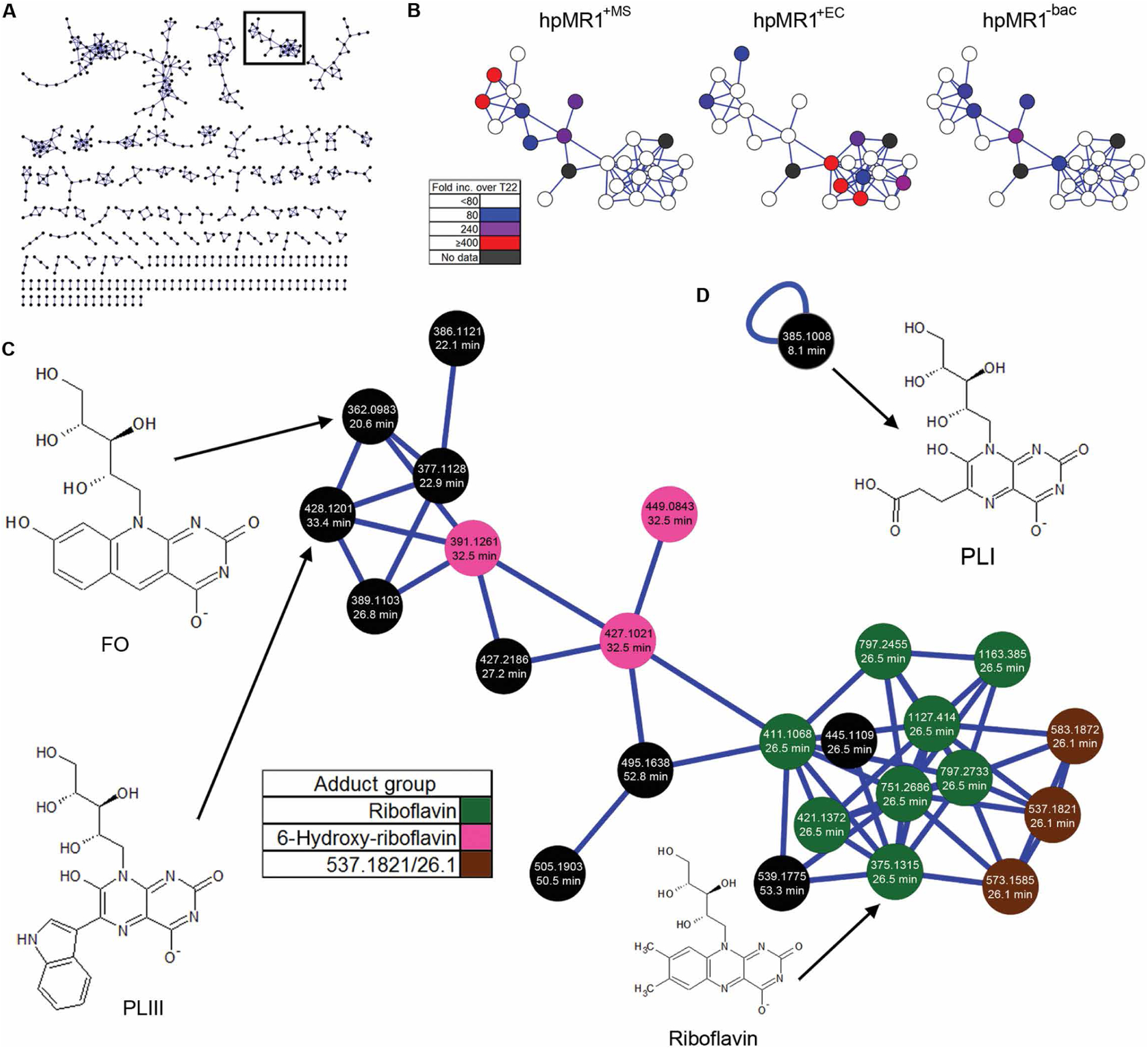
GNPS assists in the identification of novel hpMR1 eluted ions. (**A**) Molecular network of ions eluted from hpMR1 in a force-directed layout showing clusters ≥2 nodes. Each black node represents an ion MS2 fragment spectra connected by a blue edge based on spectral similarity. The black outline denotes the riboflavin cluster in (**B**) and (**C**). (B) Relative abundance of ions in the riboflavin cluster for hpMR1^+MS^ (left), hpMR1^+EC^ (middle), and hpMR1^−bac^ (right). Color indicates the fold increase over T22 in each respective hpMR1 sample. (C) Detailed image of the riboflavin network. The average ion *m*/*z* and normalized average retention time for each ion are indicated. Green nodes, riboflavin adducts; pink nodes, adducts of ion 391.1261/32.5; brown nodes, adducts of ion 537.1821/26.1. (**D**) Single nonclustering node associated with PLI (385.1008/8.1).

To facilitate identification of the novel ligands from our LC-MS experiments, we subjected the negative ion polarity spectra to Global Natural Products Social Molecular Networking (GNPS) analysis, which clusters compounds with similar MS2 fragment spectra and molecular structures (19). Overall, we identified 154 clusters with anywhere from 2 to 48 nodes per cluster (Fig. 5A), with 1069 ions that could not be clustered. No ions clustered with rRL-6-CH_2_OH/5-OP-RU, and RL-6-Me-7-OH clustered with only one other ion, which we identified as acetylated RL-6-Me-7-OH (fig. S4A). One large cluster contained two of the most distinct ions for hpMR1^+MS^ (Fig. 5B). Database spectral matching identified riboflavin and several riboflavin adducts or derivatives as constituents of this cluster (Fig. 5C, table S1, and figs. S5 and S6). Using mass accurate database searches and comparing fragment spectra, we identified three additional MR1T ligands in this cluster. The most prevalent hpMR1^+MS^-specific ion in the cluster was identified as 7,8-didemethyl-8-hydroxy-5-deazariboflavin (FO) (Fig. 5C and fig. S6) (20). An ion near FO in the riboflavin cluster and present in both hpMR1^+EC^ and hpMR1^+MS^ was identified as 6-(1*H*-indol-3-yl)-7-hydroxy-8-ribityllumazine or photolumazine III (PLIII) (Fig. 5C and fig. S6) (21). Another ion unique to hpMR1^+MS^ had similar fragments and neutral loss ions as PLIII and RL-6-Me-7-OH but did not cluster with riboflavin. We identified this ion as 6-(2-carboxyethyl)-7-hydroxy-8-ribityllumazine or photolumazine I (PLI) (Fig. 5D and fig. S6) (21). These findings demonstrate that unique MR1 ligands can be formed in the context of infection with mycobacteria. In addition to riboflavin, there was one additional spectral match in the MR1 ligand repertoire, which we identified as hesperidin, a non-uracil–based compound. LC-MS of synthetic hesperidin confirmed the initial spectral match by GNPS (fig. S7). Although this molecule was detected in all MR1 preparations, including hpMR1^−bac^, it was completely missing from T22.

**Fig. 6.**
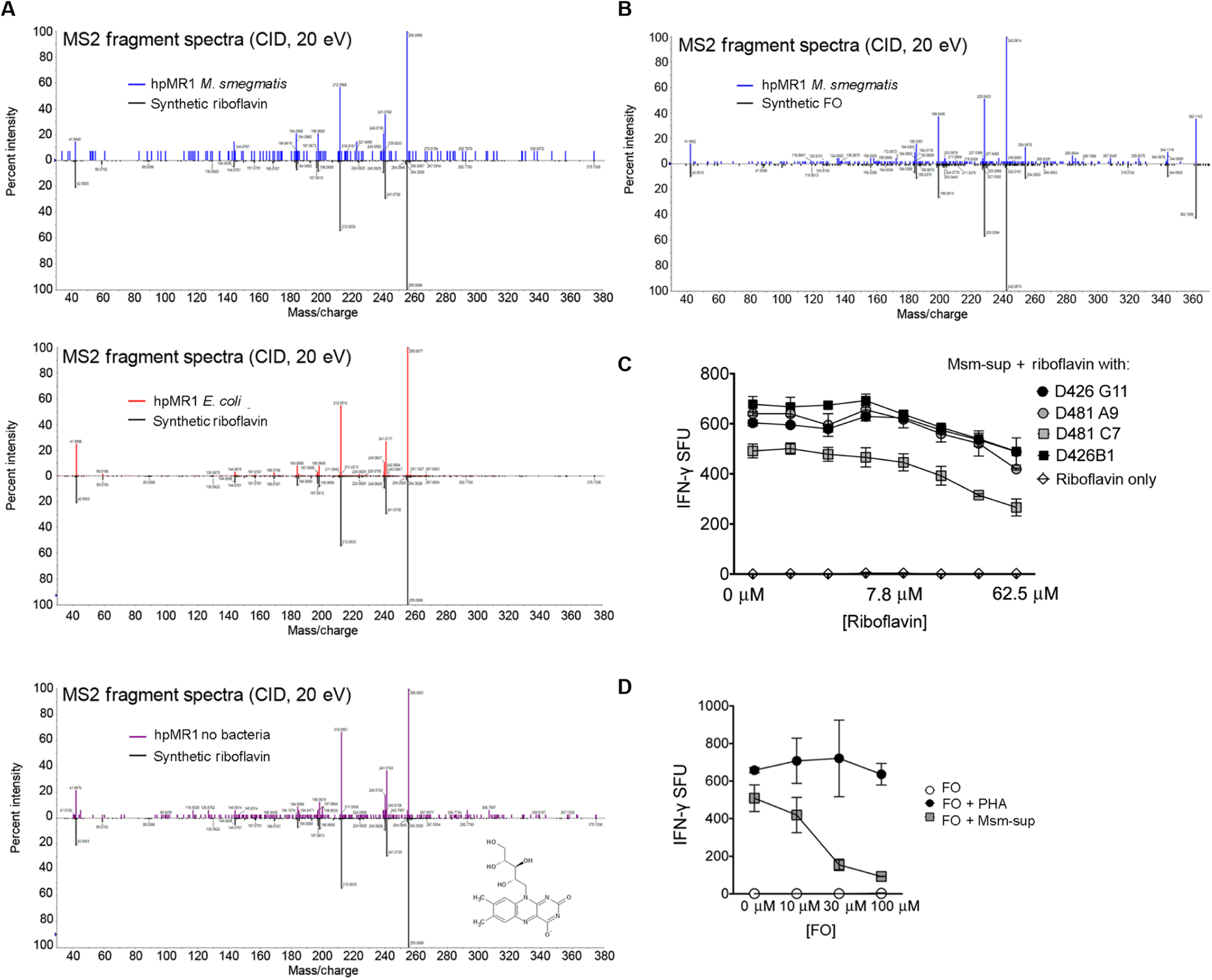
Riboflavin and FO are blocking ligands for MR1Ts. (**A**) MS2 fragment spectra of precursor ion in the indicated sample, indicating that synthetic riboflavin matches the eluted ion in hpMR1^+EC^, hpMR1^+MS^, and hpMR1^−bac^. (**B**) MS2 fragment spectra of precursor ion in the indicated sample, indicating that synthetic FO matches the eluted ion in hpMR1^+MS^. CID, collision-induced dissociation. (**C** and **D**) MR1T clone responses to BEAS-2B cells incubated with riboflavin (C) (7.8 to 500 μM) or FO (D) (10, 30, or 100 μM) before the addition of Msm-sup. Phytohemagglutinin (PHA) was used as a control for toxicity. Results in (C) and (D) are representative of three independent experiments. Error bars represent means and SD from technical replicates.

To confirm the identification of novel ligands, we purchased riboflavin and hesperidin and synthesized FO, PLI, and PLIII (fig. S8). With all five compounds, the observed masses of the eluted ion were within 5 ppm (parts per million) of the corresponding observed synthetic ion and the theoretical mass (figs. S5 and S6). Further, the MS2 fragment spectra for the synthetic ligands matched the spectra of the ion eluted from the hpMR1^+EC^ and/or hpMR1^+MS^, confirming their identification as MR1 ligands (Fig. 6, A to D).

### Riboflavin and FO are inhibitory ligands for MR1Ts, whereas PLI and PLIII are activating

**Fig. 7.**
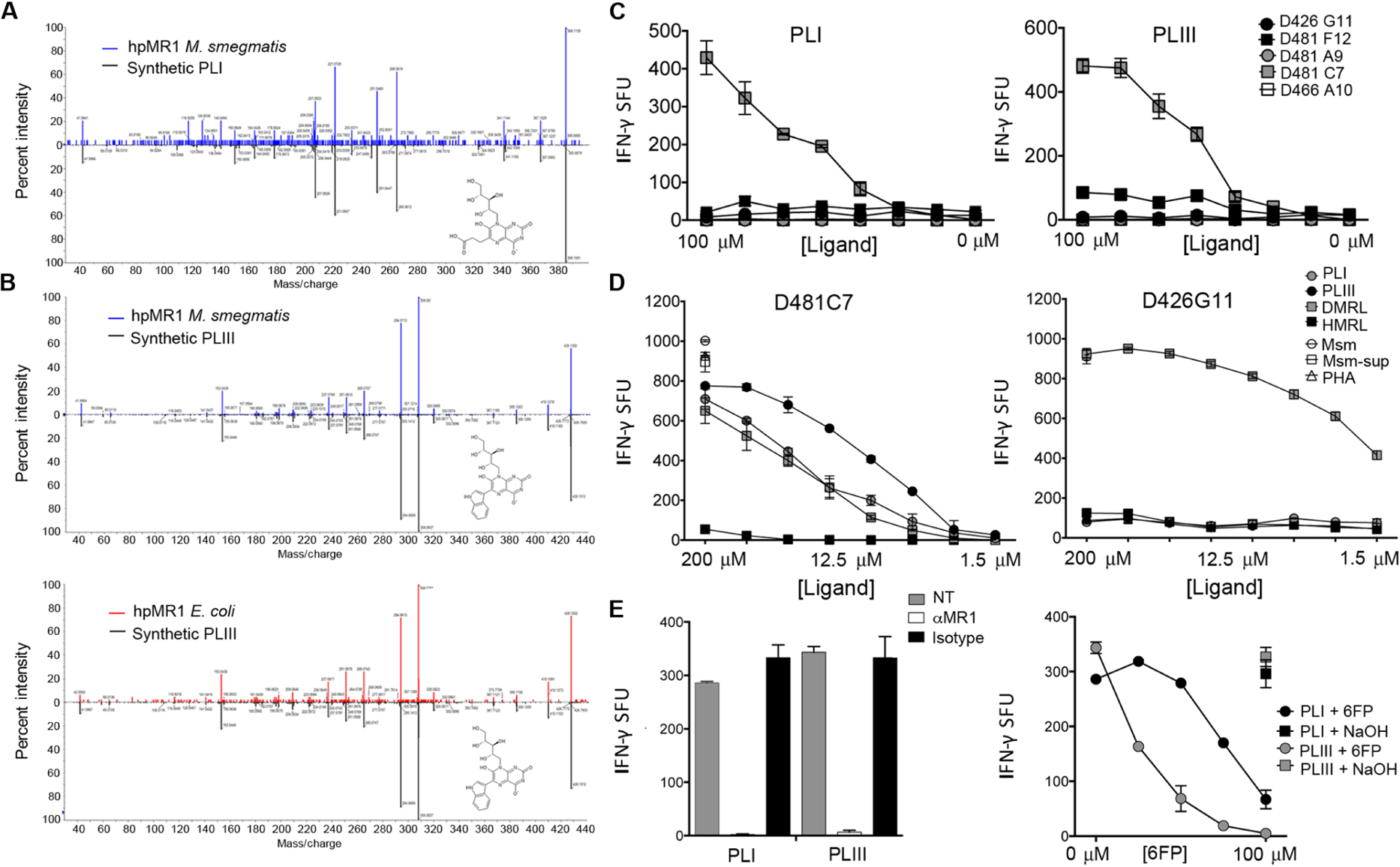
PLI and PLIII are activating ligands for MR1Ts. (**A**) MS2 fragment spectra of precursor ion in the indicated sample, indicating that synthetic PLI matches the eluted ion in hpMR1^+MS^. (**B**) MS2 fragment spectra of precursor ion in the indicated sample, indicating that synthetic PLIII matches the eluted ion in hpMR1^+EC^ and hpMR1^+MS^. (**C**) MR1T clone responses to DC pulsed with PLI or PLIII at the indicated concentration. (**D**) MR1 cell clone D481C7 or D426G11 responses to DC incubated with synthetic PLI, PLIII, RL-6,7-diMe (DMRL), or RL-6-Me-7-OH (HMRL), at 1.56 to 200 μM, or *M. smegmatis* (Msm), Msm-sup, or PHA. (**E**) Left: The D481C7 MR1T clone response to DC pulsed with PLI or PLIII was blocked with the anti-MR1 blocking antibody. Right: 6-FP was added at increasing concentrations to DC before pulsing with PLI or PLIII. Results in (C) and (D) are representative of three independent experiments. Error bars represent means and SD from technical replicates.

To determine whether riboflavin, hesperidin, FO, PLI, and PLIII were MR1 antigens, we tested the synthetic molecules for their ability to stimulate MR1T clones. Neither riboflavin nor FO was capable of MR1T activation (Fig. 7, A and B). To determine whether riboflavin or FO antagonizes MR1T activation like 6-formylpterin (6-FP) (22, 23), we preloaded the antigen-presenting cells (APCs) with riboflavin or FO and then tested them for their ability to present antigens from *M. smegmatis* supernatant (Msm-sup) to MR1Ts. Preincubation of APC with both riboflavin and FO blocked the MR1T clone response to Msm-sup (Fig. 7, C and D). In contrast to riboflavin and FO, PLI and PLIII were antigenic. To determine whether the antigenic responses were a reflection of the relative potency of each antigen, we tested PLI and PLIII for their reactivity to MR1T clones with distinct TCRs across a broad range of ligand concentrations (Fig. 7, E and F). PLI and PLIII were activating ligands for the D481C7 MR1T clone (Fig. 7E), with responses similar to RL-6,7-diMe (Fig. 7F). There were modest responses to PLI and PLIII for the D462E4 and D481F12 MR1T clones, and no response by the D481A9 or D426G11 clones at any of the doses, despite the ability of these clones to recognize RL-6,7-diMe, as shown for D426G11 (Fig. 7F). To validate that the response to PLI and PLIII by the D481C7 clone was restricted by MR1, we demonstrated that IFN-γ release was blocked by incubation with 6-FP or the anti-MR1 blocking antibody (Fig. 7G). Although hesperidin was confirmed by MS analysis of the synthetic compound to be a ligand eluted from MR1, it was not antigenic or antagonistic (fig. S7).

### MR1T clones with distinct TCR usage selectively recognize activating ligands

**Fig. 8.**
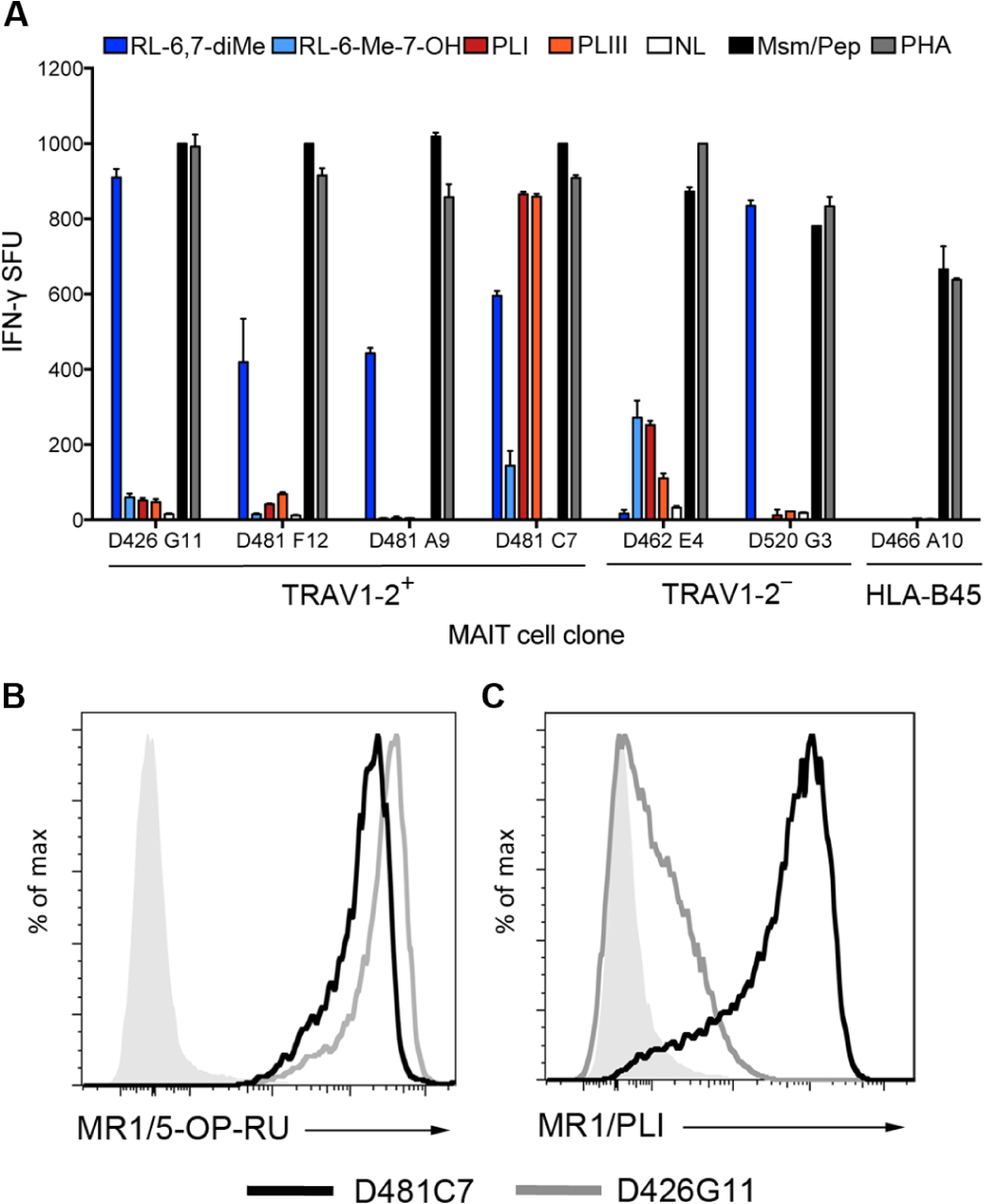
MR1T clones with distinct TCR usage display differential recognition of discrete activating ligands. (**A**) MR1T clones using the TRAV1–2^+^ or TRAV1–2^−^ α-chain, or an HLA-B45–restricted T cell clone (D466 A10), responses to DC incubated with 100 μM PLI, PLIII, RL-6,7-diMe, or RL-6-Me-7-OH. *M. smegmatis* (Msm) for MR1T clones and the CFP10_2–9_ peptide (Pep) for the HLA-B45 clone were used as positive controls. NL indicates the no ligand control condition. (**B**) hpMR1 loaded with PLI was generated and used to stain the D481C7 and D426G11 clones to demonstrate that distinct TCR-diverse MR1T clone responses to PLI correspond to tetramer staining. Gray histograms are MR1/PLI tetramer staining of a control HLA-A2–restricted CD8^+^ T cell clone. Results are representative of three independent experiments. Error bars represent means and SD from technical replicates.

Given the large differences in response between D481C7 and D426G11, we postulated that discrete TCR usage could be associated with ligand discrimination. To examine this, we tested the TCR-diverse MR1T clones for their ability to recognize each antigen. Every clone responded robustly to APC infected with *M. smegmatis*; however, large differences were observed in response to the novel and known ligands (Fig. 8A). For example, although the D481F12 and D481A9 MR1T clones responded to RL-6,7-diMe, they had modest or no recognition of RL-6-Me-7-OH, PLI, and PLIII. In contrast, the D481C7 responded to RL-6-Me-7-OH as well as RL-6,7-diMe, PLI, and PLIII. Because TRAV1–2^−^ cells were stained with the hpMR1^+EC^ tetramer, we also tested the ability of a TRAV1–2^−^ clone described in Fig. 2, as well as a recently described TRAV12–2 MR1T clone (12), to respond to these ligands. Although the TRAV1–2^−^ D520G3 clone did not respond to PLI or PLIII, the D462E4 TRAV12–2 MR1T clone had comparable responses with RL-6-Me-7-OH and PLI that were greater than PLIII (Fig. 8A). The TRAV12–2 MR1T clone did not respond to RL-6,7-diMe, as previously reported (12). For clones D426G11 and D481C7, the magnitude of the *M. smegmatis* response was comparable with RL-6,7-diMe or PLI/PLIII, respectively, confirming the identification of an optimal activating antigen for that TCR. In contrast, the modest responses observed for clones D481A9, D481F12, and D462E4 suggest that the optimal antigen for those clones remains to be found. Finally, to confirm MR1 restriction and validate the ability of TCR-diverse MR1Ts to distinguish ligands, we refolded hpMR1 and exogenously loaded it with PLI for tetramer staining. As shown in Fig. 8B, the D426G11 and D481C7 MR1 clones have equal staining with the MR1/5-OP-RU tetramer. In contrast, although the D481C7 clone was equally stained with the MR1/PLI and MR1/5-OP-RU tetramers, there was only minimal staining with the MR1/PLI tetramer for the D426G11 clone (Fig. 8C). Together, these data demonstrate that discrete MR1T TCRs can distinguish between distinct MR1 antigens.

## DISCUSSION

Within the immune system, T cells respond to perturbations in the intracellular environment. Broadly speaking, conventional MHC-I and MHC-II sample the peptidome, through the processing and presentation of protein antigens, and CD1 samples the microbial cell wall through the processing and presentation of lipids and glycolipids. MR1 is unique in its ability to present microbial small-molecule metabolites, and a role for riboflavin biosynthesis and the riboflavin biosynthetic intermediate 5-A-RU has been established (6, 10, 11, 24). The Rossjohn and McCluskey groups have demonstrated that modification of 5-A-RU via other small molecules such as glyoxyl or methylglyoxyl results in potent antigenic compounds such as 5-OE-RU and 5-OP-RU (10). These molecules can be observed bound to MR1 in crystal structures and have been used to generate MR1 tetramers. However, whether 5-OP-RU or 5-OE-RU is presented in the context of infection is not yet clear. At present, there is evidence supporting the recognition of a more diverse set of antigens by MR1Ts. For example, recent work has demonstrated greater diversity in the CDR3 usage of TRAV1–2^+^ MR1T TCRs and TRAV1–2^−^ MR1Ts defined by staining with the 5-OP-RU and 6-FP MR1 tetramers (4, 14), and sequencing of MR1Ts sorted on the basis of their functional response to different microbes revealed selective CDR3 usage (14). In addition, we identified and cloned an MR1-restricted, TRAV12–2^+^ T cell that, although able to bind the MR1/5-OP-RU tetramer, can recognize infection with *S. pyogenes*, a pathogen that cannot synthesize riboflavin (12). As a result, we hypothesized that MR1 can display microbial ligands not associated with riboflavin biosynthesis. To address this hypothesis, we expressed hpMR1 in insect cells in the presence of live microbes and used MS and molecular networking to evaluate the ligands. From this analysis, we observed an unexpectedly diverse array of ligands.

Using molecular networking, we could identify a subset of known MR1T ligands that are derived during riboflavin biosynthesis. We were also able to identify additional bacterial MR1T ligands including FO, PLI, and PLIII. FO is a precursor to coenzyme F_420_, a biosynthesis pathway limited to soil-based bacteria such as archaea and some actinobacteria, including *M. tuberculosis* (20, 25). FO is generated when 4-hydroxyphenylpyruvate from the tyrosine biosynthesis pathway and 5-A-RU from the riboflavin biosynthesis pathway react, catalyzed by the FO synthase enzyme. On the basis of the shared ribityllumazine structure of PLI and PLIII with RL-6,7-diMe, it is plausible that both PLI and PLIII are direct products from reactions with 5-A-RU and molecules from other metabolic pathways. For example, PLI could be a secondary metabolite generated through a spontaneous reaction between α-ketoglutarate and 5-A-RU. In mycobacteria, glutamate dehydrogenase activity is necessary for the generation of α-ketoglutarate and depends on the availability of nitrogen species such as ammonium (26). Hence, nitrosative stress occurring during intracellular infection could contribute to the generation of PLI and could explain why PLI was only observed in the case of infection with the intracellular mycobacterial microbe. In this regard, even if 5-A-RU is central, it demonstrates that MR1 samples alternate metabolic pathways in the setting of intracellular infection. The extent to which these ligands are associated with MR1T expansion and activation remains to be explored.

A significant finding from the molecular networking result is the number of molecules that did not cluster with ligands deriving from 5-A-RU or riboflavin metabolism. These clusters suggest that there are families of structurally distinct bacterially derived ligands because they do not share fragment patterns with those that have been previously described. Here, we identified hesperidin as a non-uracil–based ligand. The relatively low abundance of the known ligands in the hpMR1^+MS^ compared with hpMR1^+EC^ despite similar recognition by diverse MR1T clones supports this diversity. Other groups have also recently described non-uracil–based MR1T ligands. For example, Keller *et al*. (27) described a series of drug and drug-like molecules with chemically diverse structures that can bind MR1. A limitation of our approach is that we have been unable to anchor many of the molecular clusters so that many of the chemical structures of ions specific to MR1 loaded in the context of bacterial infection remain to be determined.

Our MS and molecular networking results demonstrated the diversity of MR1-presented ligands; however, the functional importance of these ligands was not clear. To establish the relevance of diverse ligand display to MR1Ts, we developed tetramers derived from the bacterially loaded MR1 monomers. Unlike the MR1/5-OP-RU tetramer, this process of ligand loading produces MR1 protein loaded with a heterogeneous mixture of ligands derived from the bacteria, the medium, and the insect cells. Because not all of these MR1 monomers are associated with TCR binding, the tetramers likely have less avidity than those loaded with a single ligand. We note that the MR1/5-OP-RU tetramer equally stains all of the diverse TCR clones used in this report despite different functional avidity. As a result, the MR1/5-OP-RU tetramer does not serve to fully define the ligand specificity of these clones. This parallels the observation that αGalCer CD1d tetramers stain all type I natural killer T cells, masking underlying ligand discrimination (28). In contrast, tetramers made with hpMR1 loaded in the context of microbial infection stained a distinct population of MR1Ts. The synthesis and functional evaluation of PLI and PLIII provide confirmation of this hypothesis, as T cell clones with different TCRs responded differentially to these ligands compared with the known ligands RL-6,7-diMe and RL-6-Me-7-OH, indicating that these TCRs can discriminate between seemingly similar ligands.

The identification of additional MR1 ligands recognized by distinct MR1T TCRs extends previous reports on the unexpected diversity in TCR usage among microbe-reactive MR1Ts. Together, these data suggest that MAIT cell expansion is influenced by microbial MR1/ligand exposure. Whether these selective expansions persist after removal of antigen remains to be determined. For example, although H2-M3–restricted T cell populations persist and resemble classical memory cells after primary infection, they do not reexpand upon secondary exposure (29). However, if selective and persistent microbial-associated expansions occur, then this would support the hypothesis that MAIT cells have immunological memory much like conventional T cells. One unexpected result was the observation that both *E. coli* and mycobacteria generate inhibitory and activating MR1 ligands. The ability of bacteria to generate inhibitory MR1 ligands suggests that MR1T recognition may reflect a balance between these competing ligands. Our work also raises the question of the molecular basis for the selective ligand recognition observed here, which will require future structural analysis. These findings imply that the selective expansion or maintenance of MR1Ts selective to the microbial metabolome could be harnessed in immunotherapeutic or vaccination strategies. In this regard, determining whether MR1Ts have immunologic memory, defined by expansion and retention of antigen-selective T cells, will be critical.

## MATERIALS AND METHODS

### Study design

The objectives of this study were to identify MR1T ligands presented in the context of microbial infection and to evaluate activation of TCR-diverse MR1Ts by these ligands. To enable these goals, we designed and performed experiments in cellular immunology, protein biochemistry, and MS. The number of independent experiments is outlined in the figure legends, where applicable.

### Human subjects

This study was conducted according to the principles expressed in the Declaration of Helsinki. Study participants, protocols, and consent forms were approved by the Institutional Review Board at Oregon Health & Science University (OHSU) (IRB00000186).

### Cells and bacteria

MR1T clones were expanded and maintained as previously described (3, 12). Hi5 insect cells were used for expression of hpMR1, and *E. coli* or *M. smegmatis* (strain mc^2^155) were used for co-infection of Hi5 cells. PBMCs were isolated from whole blood obtained by apheresis with informed consent, as previously described (30). Human monocyte-derived dendritic cells (DCs) were isolated from PBMCs, as previously described (31). BEAS-2B cells were obtained from the American Type Culture Collection and cultured as recommended.

### hpMR1 production and tetramerization

hpMR1 was expressed in Hi5 cells using a baculoviral system and purified as described in Supplementary Materials and Methods. Briefly, we co-infected the insect cell culture with live bacteria and harvested the cell supernatant for purification of hpMR1 protein. This protein was then used for MS analysis and for making tetramers. Streptavidin-R-phycoerythrin–conjugated hpMR1 tetramers were used for flow cytometry, and traptavidin-conjugated hpMR1 tetramers were used for tetraSPOT analysis.

### LC-MS analysis of hpMR1

hpMR1 molecules expressed in the presence of *E. coli*, *M. smegmatis*, medium only, or T22 were analyzed as described in Supplementary Materials and Methods. Briefly, hpMR1 molecules were injected for low-pH reversed-phase nanoscale LC-MS. Ion spectra were collected using an AB Sciex TripleTOF 5600 mass spectrometer. Data were acquired in data-dependent acquisition mode with a survey mass/charge ratio (*m*/*z*) range of 150 to 1500 in negative or positive ion polarity. Extracted ion chromatograms, MS1 survey spectra, and MS2 fragment spectra were made using PeakView 1.2 (Sciex).

### Comparative analysis of eluted hpMR1 ions

All comparative analyses were completed using MarkerView 1.2.1 (Sciex) and are described in detail in Supplementary Materials and Methods. To prioritize ions for identification of new ligands, we set a series of strict thresholds. An ion with an intensity in any one of the hpMR1 samples ≥80-fold increase over the intensity of T22 was considered over background and a putative hpMR1 ligand. Within the hpMR1 ligands, if the ion intensity in either hpMR1^+MS^ or hpMR1^+EC^ was ≥10-fold increase over hpMR1^−bac^, then the ion was considered a putative bacterial-derived ligand. All other ions with intensities <80-fold increase over T22 were considered background ions. Molecular networking was completed using the GNPS workflow (19), as described in Supplementary Materials and Methods.

### Synthesis of synthetic ligands

The following protocols were followed for synthesis of all ligands: For air- and water-sensitive reactions, glassware was oven-dried before use, and reactions were performed under argon. Dichloromethane, dimethylformamide, and tetrahydrofuran were dried using the solvent purification system manufactured by Glass Contour Inc. All other solvents were of American Chemical Society (ACS) grade (Fisher Scientific) and used without further purification unless otherwise indicated. Analytical thin-layer chromatography was performed with silica gel 60 F_254_ glass plates (SiliCycle). Flash column chromatography was conducted with either prepacked RediSep Rf normal/reverse phase columns (Biotage). High-performance liquid chromatography (HPLC) was performed on a Varian ProStar 210 (Agilent) with a flow rate of 20 ml/min using Polaris 5 C18-A columns (150 mm × 4.6 mm, 3 m analytical; 150 mm × 21.2 mm, 5 m preparative) (Agilent). HPLC analytical conditions were as follows: mobile phase (MP) A: 0.1% formic acid (aq); MP B: 0.1% formic acid in acetonitrile; flow rate, 1.0 ml/min; condition A: 0 to 2 min, 0%B; 2 to 15 min, 0 to 100%B; 15 to 17 min, 100%B; condition B: 0 to 1 min, 0%B; 1 to 12 min, 0 to 40%B; 12 to 13 min, 40%B; 13 to 14 min, 0%B; ultraviolet (UV)–visible detection: λ_1_ = 254 nm, λ_2_ = 280 nm. All final products were ≥95% purity as assessed by this method. Retention times (*t*_R_) and purity refer to UV detection at 220 nm. Specific details for the synthesis of FO, PLI, and PLIII are described in Supplementary Materials and Methods and in fig. S8.

### ELISPOT assays

For the plate-bound tetramer ELISPOT (tetraSPOT) assay, ELISPOT plates were coated with an anti–IFN-γ antibody, as previously described (32). At the time of coating, MR1 tetramers generated from uninfected, *E. coli*–infected, or *M. smegmatis*–infected cells, as described above, were also added to wells at concentrations between 0 and 500 nM per well. After overnight incubation at 4°C, ELISPOT plates were washed three times with phosphate-buffered saline and then blocked with RPMI 1640 + 10% human serum for 1 hour. MAIT cell clones (2 × 10^4^) or whole PBMCs (5 × 10^5^) were added to wells overnight. IFN-γ ELISPOTs were enumerated following development as previously described (32). ELISPOT analysis of MR1 ligands was performed as previously described (32) and is described in Supplementary Materials and Methods.

### Flow cytometry

PBMCs were treated with 50 nM dasatinib (Axon Medchem) for 30 min at room temperature. Dasatinib-treated cells were stained with hpMR1^−UI^, hpMR1^−EC^, or hpMR1^−MS^ tetramers at the indicated concentrations for 1 hour at room temperature. As a comparison, cells were stained with the MR1/5-OP-RU and MR1/6FP tetramers. Cells were then washed, stained with the LIVE/DEAD Fixable Dead Cell Stain Kit (Life Technologies), and surface-stained with the antibodies listed in table S1 for 30 min at 4°C. Samples were fixed with 1% paraformaldehyde, and acquisition was performed using a Fortessa flow cytometer with FACSDiva software (BD Biosciences). All flow cytometry data were analyzed using FlowJo software (TreeStar) and Prism (GraphPad). Gating strategies are shown in fig. S2.

### Isolation of TRAV1–2^−^ T cell lines and clones

TRAV1–2^−^ T cell clones were generated from CD8^+^, CD4^−^, γδ TCR^−^, TRAV1–2^−^ T cells, as described in Supplementary Materials and Methods. Clonality was determined through uniformity of flow cytometry staining based on cell surface phenotype and uniformity in functional response by IFN-γ ELISPOT.

### Expansion of T cell clones

T cell clones were cultured in the presence of x-rayed (3000 cGy using X-RAD320, Precision X-Ray Inc.) allogeneic PBMCs, x-rayed allogeneic LCL (6000 cGy), and anti-CD3 monoclonal antibody (20 ng/ml; Ortho-clone OKT3, eBioscience) in RPMI 1640 medium with 10% human serum in a T-25 upright flask in a total volume of 30 ml. The cultures were supplemented with interleukin-2 on days 1, 4, 7, and 10 of culture. The cell cultures were washed on day 5 to remove soluble anti-CD3.

### Inclusion body production and refolding, loading, and tetramerization of hpMR1

Inclusion body production and the refolding protocol were based on previously described methods from Kjer-Nielsen *et al*. (6). To confirm a successful loading protocol with refolded hpMR1, we first loaded with 5-OP-RU, as described in Supplementary Materials and Methods. For loading with PLI, a 100-fold molar excess of PLI was used. Loaded protein was then tetramerized as described above.

### Statistical analysis

For statistical comparisons of PBMC donors, normality was assessed using the Shapiro-Wilk test. In cases where the data were not normally distributed, statistical comparisons for PBMC donors were performed using a Wilcoxon matched-pairs signed-rank test using GraphPad Prism. In cases where the data were normally distributed, statistical comparisons for PBMC donors were performed using a paired *t* test using GraphPad Prism.

## Supplementary Material

Supplemental material

Table S1
