# Supplementary material for: MR1 displays the microbial metabolome driving selective MR1-restricted T cell receptor usage
Source: Sci Immunol. Author manuscript; Available in PMC 2020 Mar 21. (PMC7085347; doi:10.1126/sciimmunol.aao2556)
Supplement: Supplemental material [file NIHMS1558326-supplement-Supplemental_material.pdf]

Science Immunology
AAAS

immunology.sciencemag.org/cgi/content/full/3/25/eaao2556/DC1

Supplementary Materials for

MR1 displays the microbial metabolome driving selective MR1-restricted T cell receptor usage

Melanie J. Harriff, Curtis McMurtrey, Cara A. Froyd, Haihong Jin, Meghan Cansler, Megan Null, Aneta Worley, Erin W. Meermeier, Gwendolyn Swarbrick, Aaron Nilsen, Deborah A. Lewinsohn, William Hildebrand, Erin J. Adams, David M. Lewinsohn*

*Corresponding author. Email: lewinsod@ohsu.edu

Published 13 July 2018, *Sci. Immunol.* **3**, eaao2556 (2018)
DOI: 10.1126/sciimmunol.aao2556

The PDF file includes:

Materials and Methods
Fig. S1. Expression and validation of hpMR1.
Fig. S2. hpMR1 tetramer staining of MAIT cell clones.
Fig. S3. MR1T clone responses to 5-OP-RU in tetraSPOT assay with MR1/5-OP-RU.
Fig. S4. Raw LC-MS data for previously identified ligands 5-OP-RU/rRL-6-CH_2OH and RL-6-Me-7-OH.
Fig. S5. Raw MS2 fragment spectra for acetyl RL-6-Me-7-OH and riboflavin adducts.
Fig. S6. Raw LC-MS data for newly described MR1 ligands riboflavin, FO, PLI, and PLIII.
Fig. S7. LC-MS, MS2, and MR1T activation data for hesperidin.
Fig. S8. Chemical synthesis pathway for FO, PLI, and PLIII.
References (*33*–*35*)

Other Supplementary Material for this manuscript includes the following:

(available at immunology.sciencemag.org/cgi/content/full/3/25/eaao2556/DC1)

Table S1 (Microsoft Excel format). Tabulated raw data.

Materials and Methods

Human Subjects. This study was conducted according to the principles expressed in the Declaration of Helsinki. Study participants, protocols, and consent forms were approved by the Institutional Review Board at Oregon Health & Sciences University (IRB00000186).

Cells and Bacteria. The D426B1, D481F12, D481A9, and D481C7 MR1-restricted T cell clones were expanded and maintained as previously described [3]. All of these T cell clones express TRAV1-2 and recognize pathogen-infected cells in an MR1-restricted manner. Isolation and expansion of the TRAV12-2 D462E4 MR1-restricted T cell clone was recently described [12]. D466A10 is an HLA-B45-restricted T cell clone that responds to the $CFP10_{2\text{-}9}$ peptide from the *M. tuberculosis* CFP10 protein. Hi5 insect cells were used for expression of hpMR1, and *E. coli* or *M. smegmatis* (strain mc^2155) were used for co-infection of Hi5 cells. Peripheral blood mononuclear cells (PBMC) from unexposed donors, donors with latent tuberculosis infection (LTBI), or active TB were isolated from whole blood obtained by apheresis with informed consent as previously described [30]. Human monocyte-derived dendritic cells (DC) were isolated from PBMC by plate adherence and cultured in RPMI+10% heat-inactivated (hi)-HuS with 30 ng/ml GM-CSF (Immunex) and 10 ng/ml IL-4 (Immunex) as previously described [31]. BEAS-2B cells were obtained from ATCC and cultured in DMEM+10% hi-fetal bovine serum.

MR1 production/tetramerization. Human platform MR1 (hpMR1) was expressed in Hi5 cells using a baculoviral system, harvesting the cell supernatant between 68-72 hours after infection. To incorporate bacterial ligands into hpMR1 during production, we co-infected the insect cell culture with live bacteria. Between 18-20 hours after adding the baculovirus to the insect cells, we added live *E. coli* (at 4×10^1 BL21 *E. coli* cells/mL insect cell culture) or *M. smegmatis* (at 4×10^3 *M. smegmatis* cells/mL insect cell culture) to the insect cell culture. The cell supernatant was harvested between 68-72 hours after infection with baculovirus as with expression of hpMR1 in the absence of bacteria.

The cell supernatant was concentrated and buffer exchanged twice with 10 mM HEPES, pH 7.2, 150mM NaCl (HBS), with the final volume being between 200-400 mL. The protein was then centrifuged to pellet any remaining cell debris. Imidazole was added to the protein to 40 mM, then Ni-NTA resin was added, and the protein was bound overnight at 4°C. The Ni-NTA resin was collected, and the protein was eluted with 200 mM imidazole in HBS, followed by 400 mM imidazole in HBS. Protein was treated with the 3C protease, the imidazole was diluted to approximately 40 mM, and protein was applied to Ni-NTA resin, followed by a wash with HBS to separate the 3C-treated hpMR1 (lacking the His tag) from proteins

that were still able to bind Ni-NTA. 3C-treated hpMR1 was then concentrated and buffer exchanged 3 times into 10 mM Tris, pH 8.0, 50 mM NaCl. The protein was then biotinylated overnight at 4°C using the BirA enzyme. The protein was then purified over an S200 column in HBS. This protein was then used for making tetramers and mass spectrometry analysis. Tetramers were made by incubating biotinylated hpMR1 protein with a 1.1 molar excess of either phycoerythrin-conjugated streptavidin (SA-PE) (Invitrogen) or traptavidin [33] for every 4 moles of hpMR1. SA-PE-conjugated tetramers were used for flow cytometry, and traptavidin-conjugated tetramers were used for tetraSPOT analysis.

LCMS analysis of hpMR1. A total of 10 µg of hpMR1 expressed in the presence of *E. coli*, *M. smegmatis*, media only, or T22 was injected for low pH reverse-phase nano-scale LCMS. Before injection, 5 peptides (RLDDDGNFQL, ATWAENIQV, YTMDGEYRL, SIGGVFTSV, and SLFGQRIEV) were added to the MR1 at a concentration of 400µM to act as standards to normalize retention times between injections. Nano-LCMS was performed using an Eksigent nano-LC-4000 with an Eksigent autosampler (Sciex). Mobile phase solvents were: solvent A (98% water, 2% acetonitrile, 0.1% formic acid), and solvent B (95% acetonitrile, 5% water, 0.1% formic acid). Whole hpMR1 or T22 was loaded onto a C18 trap column (350 µm [i.d.] × 0.5 mm long; ChromXP) and desalted at 5 µl/min for 5 minutes using 100% solvent A. After desalting, the trap was placed in line with a C18 separation column (75 µm [i.d.] × 15 cm long; ChromXP). Sample was then eluted at 300 nl/min using two linear gradients: 1. 2% solvent B to 40% solvent B in 70 minutes, 2. 40% solvent B to 80% solvent B in 7 minutes with a 20 minute recalibration in 2% solvent B. In between replicates, the trap and separation columns were replaced. Eluate was ionized using a Nanospray III ion source (Sciex). Ion spectra were collected using an AB Sciex TripleTOF 5600 mass spectrometer. Data was acquired in data-dependent acquisition mode with a survey m/z range of 150-1500 in negative or positive ion polarity. Extracted ion chromatograms, MS1 survey spectra, and MS2 fragment spectra were made using PeakView 1.2 (Sciex). For 5-OP-RU, RL-6-Me-7-OH, FO, PLI, PLIII, and riboflavin, synthetic compounds were run under the same conditions and the hpMR1 samples. In every case, the eluted ion and synthetic: 1. Had retention times within 3 minutes of each other, 2. MS1 ions were within 3ppm of each other, 3. Had similar MS2 fragment spectra.

Comparative analysis of eluted hpMR1 ions. All comparative analyses were completed using Markerview 1.2.1 (Sciex). First, MS1 extracted ion intensities were determined using the area-underneath-the curve approach. Retention times were normalized using the standard peptides added to the hpMR1 sample as previously mentioned. A retention time tolerance of 5 min and mass tolerance of 50 ppm were used to align the peaks. Comparisons (t-tests) of ion intensities were performed in Markerview.

All data was exported to and the volcano plots were made in Excel. Average intensities between replicates were used for further analysis. To prioritize ions for identification of new ligands a series of strict thresholds were set. An ion with an intensity in any one of the hpMR1 samples >= 80 fold increase over the intensity of T22, was considered over background and a putative hpMR1 ligand. Within the hpMR1 ligands, if the ion intensity in either hpMR1+MS or +EC was >=10 fold-increase over hpMR1-bac, the ion was considered a putative bacterial-derived ligand. Putative bacterial ligands are shown as blue dots. All other ions with intensities < 80-fold increase over T22 were considered background ions and shown as grey dots.

Molecular Networking. Molecular networking was completed using the Global Natural Products Social Molecular Networking (GNPS) workflow [19]. Raw LCMS *.wiff files were converted to Mascot Generic Format (*.mgf) using the AB SCIEX MS Data Converter. Converted files were then uploaded to the GNPS servers and analyzed online. First, the data was filtered by removing all MS2 peaks within +/- 17 Da of the precursor m/z. MS2 spectra were window filtered by choosing only the top 6 peaks in the +/- 50Da window throughout the spectrum. The data was then clustered with MS-Cluster with a parent mass tolerance of 2.0 Da and an MS2 fragment ion tolerance of 0.5 Da to create consensus spectra. Further, consensus spectra that contained less than 2 spectra were discarded. A network was then created where edges were filtered to have a cosine score above 0.70 and more than 10 matched peaks. Further edges between two nodes were kept in the network if and only if each of the nodes appeared in each other's respective top 10 most similar nodes. The spectra in the network were then searched against GNPS's spectral libraries. The library spectra were filtered in the same manner as the input data. All matches kept between network spectra and library spectra were required to have a score above 0.7 and at least 4 matched peaks.

Synthesis of synthetic ligands. The following protocols were followed for synthesis of all ligands: For air- and water-sensitive reactions, glassware was oven-dried prior to use and reactions were performed under argon. Dichloromethane, dimethylformamide, and tetrahydrofuran were dried using the solvent purification system manufactured by Glass Contour, Inc. (Laguna Beach, CA). All other solvents were of ACS chemical grade (Fisher Scientific) and used without further purification unless otherwise indicated. Analytical thin-layer chromatography was performed with silica gel 60 F_{254} glass plates (SiliCycle). Flash column chromatography was conducted with either pre-packed Redisep R_f normal/reverse phase columns (Biotage). High performance liquid chromatography (HPLC) was performed on a Varian Prostar 210 (Agilent) with a flow rate of 20 ml/min using Polaris 5 C18-A columns (150 x 4.6 mm, 3 m -analytical, 150 x 21.2 mm, 5 m-preparative) (Agilent). HPLC analytical conditions: mobile phase (MP) A: 0.1%

formic acid (aq), mobile phase (MP) B: 0.1% formic acid in acetonitrile (ACN); flow rate = 1.0 ml/min; condition **A**: 0 – 2 min: 0%B, 2 – 15 min: 0-100%B, 15 – 17 min: 100%B; condition **B**: 0 – 1 min: 0%B, 1 – 12 min: 0-40%B, 12 – 13 min: 40%B, 13 – 14 min: 0%B; UV-Vis detection: λ_1 = 254 nm, λ_2 = 280 nm. All final products were ≥95% purity as assessed by this method. Retention times (t_R) and purity refer to UV detection at 220 nm.

Synthesis of 8-hydroxy-10-(2,3,4,5-tetrahydroxypentyl)pyrimido[4,5-b]quinoline-2,4(3H,10H)-dione (**FO**) [34] (Fig. S8A). Compound 5-((3-((tert-butyldimethylsilyl)oxy)phenyl)amino)pentane-1,2,3,4-tetraol (**1,** 72mg, 0.2 mmol) and compound 6-chloro-2,4-dioxohexahydropyrimidine-5-carbaldehyde (**2,** 21mg, 0.12 mmol) were mixed in 1 mL *N*,*N*-dimethylformamide and heated at 130^0C for 1.5 hour. After the reaction was over, the product was purified by Pre-LC.

Synthesis of 6-(2-Carboxylethyl)-7-oxo-8-D-ribityllumazine (PLI, **6**) [35] (Fig. S8B). 5-Nitro-6-(D-ribityl)aminouracil (**3**) (30 mg, 0.1 mmol) was dissolved in water (1 mL) and hydrogenated under H_2 atmosphere for 6 h in the presence of Pd on activated carbon (3 mg, 10%). TLC was used to detect the reaction. After TLC showed that the reaction was completed, acetic acid (0.3 mL) and 2-ketoglutaric acid (**5**, 80 mg, 0.54 mmol) were added, and the mixture was heated at 100 °C for 4 h. After cooling, the catalyst was removed by filtration and the filtrate was concentrated under reduced pressure. EtOH was used to wash the reaction mixture. The reaction was then concentrated *in vacuo* and the crude product was purified via preparative HPLC (MP A: 0.1% formic acid (aq), MP B: 0.1% formic acid in ACN; 0-5 min: 0-10%B, 5-8 min: 10-15%B, 8-10 min: 15-20%B, 10-12 min: 20-50%B). Fractions containing the desired product were pooled and concentrated *in vacuo* to yield the desired product.

Synthesis of 6-(2-Indoyl)-7-oxo-8-D-ribityllumazine (PLIII, **8**) [21] (Fig. S8B). 5-Nitro-6-(D-ribityl)aminouracil (**3**) (30 mg, 0.1 mmol) was dissolved in water (1 mL) and hydrogenated under H_2 atmosphere for 6 h in the presence of Pd on activated carbon (3 mg, 10%). TLC was used to detect the reaction. After TLC showed that the reaction was completed, acetic acid (0.3 mL) and 3-indolylglyoxylic acid (**7**, 12mg, 0.68 mmol) were added, and the mixture was heated at 100 °C for 4 h. After cooling, the catalyst was removed by filtration and the filtrate was concentrated under reduced pressure. EtOH was used to wash the reaction mixture. The reaction was then concentrated *in vacuo* and the crude product was purified via preparative HPLC (MP A: 0.1% formic acid (aq), MP B: 0.1% formic acid in ACN; 0-5 min: 0-10%B, 5-8 min: 10-15%B, 8-10 min: 15-20%B, 10-12 min: 20-50%B). Fractions containing the desired product were pooled and concentrated *in vacuo* to yield the desired product.

ELISPOT Assays. Plate-bound tetramer ELISPOT (tetraSPOT) assay: ELISPOT plates were coated with anti-IFN-γ antibody as previously described [32]. At the time of coating, MR1 tetramers generated from uninfected, *E. coli*-, or *M. smegmatis*-infected cells as described above were also added to wells at concentrations between 0 and 500nM per well. After overnight incubation at 4°C, ELISPOT plates were washed three times with PBS, then blocked with RPMI+10% human serum (HuS) for 1 hour. 2e4 MAIT cell clones, or 5e5 whole PBMC were added to wells overnight. IFN-γ ELISPOTs were enumerated following development as previously described [32].

ELISPOT analysis of MR1 ligands: ELISPOT plates were coated with anti-IFN-γ antibody and blocked as described above. 2e4 DC or 5e3 BEAS-2B cells were plated in the ELISPOT wells in RPMI+10% HuS. Synthetic ligands were added to the wells at concentrations ranging from 0-500⌠M as indicated for each experiment. *M. smegmatis* (Msm), supernatant from *M. smegmatis* (Msm-s), or PHA were used as positive controls. After one hour, 1-2e4 MR1T cells clones were added and the plate was incubated overnight at 37°C. For blocking experiments, Riboflavin or FO were incubated with 5e3 BEAS-2B cells in the ELISPOT plate for one hour. Msm-s was then added to each well for one hour prior to the addition of 1e4 MR1T cell clones.

Flow cytometry. PBMCs were treated with 50nM dasatinib (Axon Medchem) for 30 minutes at room temperature. Dasatinib-treated cells were stained with hpMR1-UI, -EC, or -MS tetramers at the indicated concentrations for one hour at room temperature. As a comparison, cells were stained with the MR1/5-OP-RU tetramer. The MR1/5-OP-RU tetramer technology was developed jointly by Dr. James McCluskey, Dr. Jamie Rossjohn, and Dr. David Fairlie, and the material was produced by the NIH Tetramer Core Facility as permitted to be distributed by the University of Melbourne [10]. Cells were then washed, stained with LIVE/DEAD Fixable Dead Cell Stain Kit (Lifetechnologies), and surface stained with the antibodies listed in Table S4 for 30 minutes at 4°C. Samples were fixed with 1% PFA, and acquisition was performed using a Fortessa flow cytometer with FACS Diva software (BD). All flow cytometry data was analyzed using FlowJo software (TreeStar) and Prism (Graphpad). Gating Strategies are shown in Fig. S9.

Isolation of TRAV1-2 negative T cell lines and clones. CD8+, $CD4^-$, γδ TCR^-, TRAV1-2 negative T cells were FACS sorted from $CD8^+$ enriched (EasySep Miltenyi kit) PBMCs and rested overnight in RPMI media containing 10% human serum and 0.5ng/mL IL-2. All flow cytometry sorting was performed with a BD Influx FACS machine on purity mode. Next, $1x10^6$ were added to $1x10^5$ x-rayed (3000 cGray using X-RAD320, Precision X-Ray Inc.) monocyte-derived autologous dendritic cells

infected with *M. smegmatis* (MOI three; one-hour infection) and incubated in RPMI 1640 with 10% human serum, rhIL-2 (5 ng/ml), rhIL-7 (0.5ng/ml), rhIL-15 (0.5ng/ml), and rhIL-12 (0.5ng/ml). Seven days later, T cells were removed and stained with CFSE (0.5uM for 15 minutes), and then added to $1X10^5$ irradiated (3500 rad) monocyte-derived autologous macrophages infected with *M. smegmatis* (MOI 3) and incubated with the same cytokine mixture as the prior week. The resulting T cell line was FACS sorted for CFSE-low MR1/Ag tetramer$^+$ TRAV1-2$^-$ CD8$^+$ cells. The sorted T cells were rested overnight in RPMI 1640 supplemented with 10% human serum, and 0.5ng/ml rhIL-2. Then the sorted cells were plated under limiting dilution analysis conditions. Therefore, 1/3/10 cell(s) per well was co-cultured with x-ray inactivated PBMC feeder ($150x10^5$/well) and LCL cells ($3x10^4$/well) in a 96-well round bottom plate. Cytokines were also added to the culture: IL-2 (5ng/ml), IL-12 (0.5ng/ml), IL-7 (0.5ng/ml), IL-15 (0.5ng/ml). Finally, anti-CD3 (0.03 µg/ml) was added to non-specifically expand each T cell. Plates were incubated at 37 C for 20 days before clones were harvested for analysis. Clones were only harvested from 96-well plates that met Poisson distribution thresholds for statistical clonality. Clonality was further determined through uniformity of flow cytometry staining based on cell surface phenotype and uniformity in functional response by IFN-γ ELISPOT.

Expansion of T Cell Clones. T cell clones were cultured in the presence of x-rayed (3000 cGray using X-RAD320, Precision X-Ray Inc.) allogeneic PBMCs, x-rayed allogeneic LCL (6000 cGray), and anti-CD3 mAb (20 ng/ml; Orthoclone OKT3, eBioscience) in RPMI 1640 media with 10% human serum in a T-25 upright flask in a total volume of 30 ml. The cultures were supplemented with IL-2 on days 1, 4, 7, and 10 of culture. The cell cultures were washed on day 5 to remove soluble anti-CD3.

Inclusion body production. Inclusion body production was based on previously described methods [6]. Briefly, the α chain of hpMR1 (human α1 and α2, bovine α3) containing a C-terminal Avi tag and the bovine β2m domain were expressed separately in BL21 E. coli by inducing for 4 hours to overnight with 1 mM IPTG at an OD600 of 0.6-1.0. Cells were lysed using a microfluidizer in buffer containing 50 mM Tris, pH 8.0, 1% Triton X-100, 1% Na-deoxycholate, 100 mM NaCl, 5 mM MgCl2, 10 mM DTT, and DNAse I. Inclusion bodies were pelleted by centrifugation and washed several times with wash buffer containing 50 mM Tris, pH 8.0, 0.5% Triton X-100, 100 mM NaCl, 1 mM EDTA, and 1 mM DTT. Inclusion bodies were then washed one time with wash buffer containing 50 mM Tris, pH 8.0, 1 mM EDTA, and 1 mM DTT. Inclusion bodies were then resuspended in the second wash buffer at a concentration of 60 mg/mL for the α chain and 30 mg/mL for β2m and aliquoted. Inclusion body pellets were stored at -80°C.

Refolding of hpMR1. The refolding protocol was adapted from Kjer-Nielsen, *et al.* [6]. To refold hpMR1, 60 mg of the hpMR1 α chain and 30 mg of β2m were each resuspended in 1 mL of 20 mM Tris, pH 8.0, 8 M Urea, 0.5 mM EDTA, and 1 mM DTT and heated at 50°C for 10 minutes before pelleting any remaining debris. 56-60 mg of hpMR1 α chain and 28-30 mg of β2m were added to 400 mL of refolding buffer (100 mM Tris, pH 8.5, 2 mM EDTA, 0.4 M arginine, 5 mM reduced glutathione, and 0.5 mM oxidized glutathione) and stirred overnight at 4°C in the absence of ligand. The refolding was then dialyzed in 4 L 10 mM Tris, pH 8.0, 3-4 times over 36-48 hours. The protein was then captured using DEAE resin and eluted with a salt gradient in 10 mM Tris, pH 8.0. Protein was then enzymatically biotinylated overnight at 4°C in 10 mM Tris, pH 8.0, 50-90 mM NaCl, followed by purification over a size exclusion column in TBS. (10 mM Tris, pH 8.0, 150 mM NaCl).

Loading and tetramerization of empty hpMR1. Loading of hpMR1 was done by incubating hpMR1 with the ligand in 100 mM HEPES, pH 7.2, 150 mM NaCl, for 18 or more hours at 4°C in the dark. To confirm a successful loading protocol, we loaded with 5-OP-RU by first producing 5-A-RU by incubating 0.6 mg 5-Nitro-6-(D-ribityl)aminouracil (**3**) with a 20-fold molar excess sodium dithionite in 50 uL 50 mM MES, pH 5.8, under N2 gas at 85-90°C for 4 hours. From this, a 10-fold molar excess of 5-A-RU and a 100-fold molar excess of methylglyoxal were added to the protein loading reaction. For loading with PLI, a 100-fold molar excess of PLI was used. Loaded protein was then tetramerized as described above for flow cytometry.

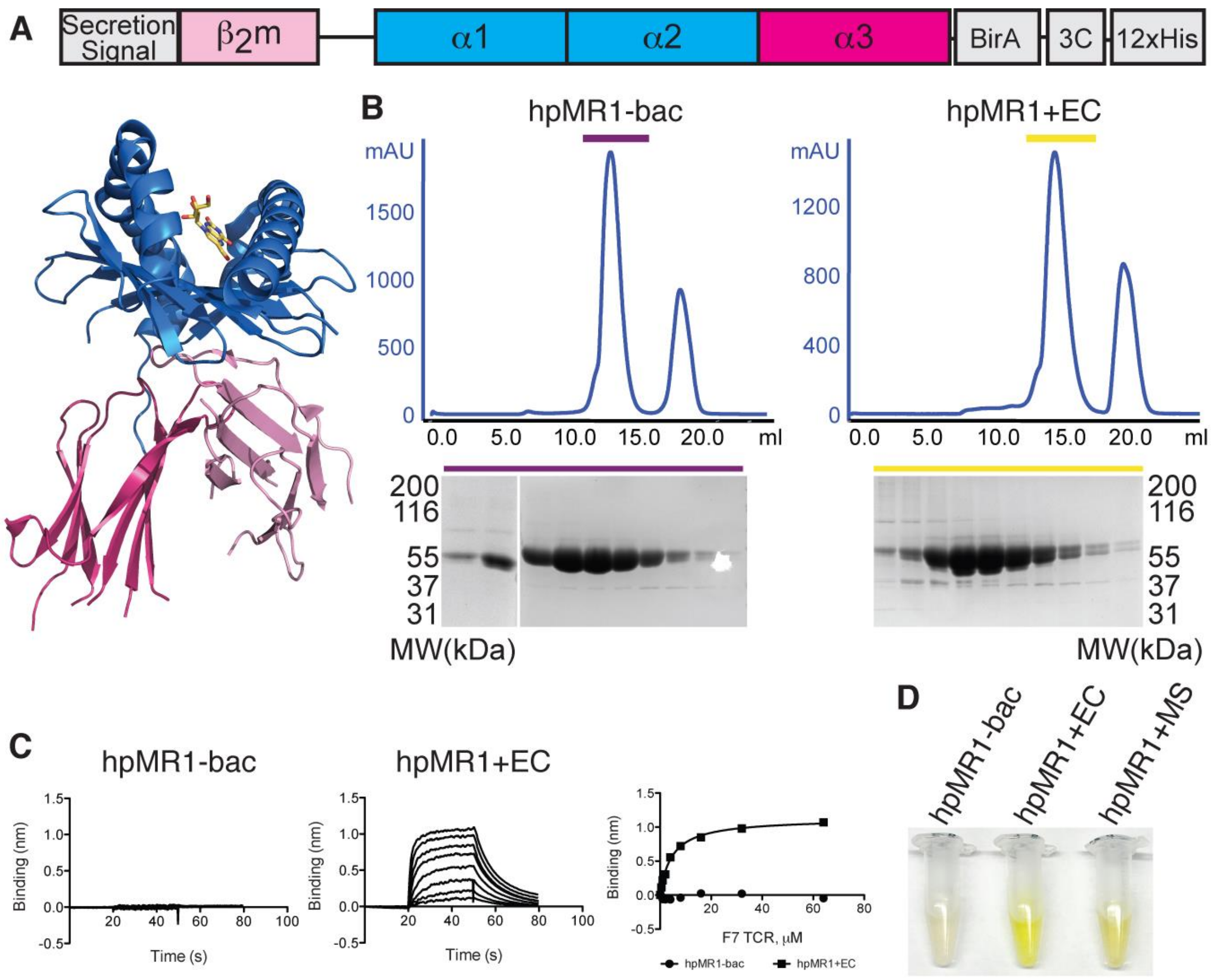

Fig. S1. Expression and validation of hpMR1. (A) Schematic representation of hpMR1 construct. Human MR1 sequence is represented in blue and bovine MR1 sequence is represented in pink. Also shown is the structure of MR1 (PDB: 4LCC) colored to match the schematic of the hpMR1 construct. (B) Size exclusion chromatography of hpMR1-bac and hpMR1+EC and SDS-PAGE of fractions from the purification. Bars represent the fractions that were collected and run on an SDS-PAGE gel. (C) Bio-layer interferometry analysis of the F7 MAIT TCR binding to hpMR1-bac or hpMR1+EC. Biotinylated hpMR1-bac or +EC was immobilized on a streptavidin biosensor followed by running increasing concentrations of the F7 MAIT TCR from 0 to 64 μM. Buffer alone (0 μM TCR) was subtracted from each, and the subtracted equilibrium binding was plotted against TCR concentration. (D) Concentrated hpMR1-bac, hpMR1+EC, and hpMR1+MS proteins after size exclusion purification are shown. Each protein has a distinctly different color, likely due to the different repertoire of ligands present from the specific bacteria or absence of bacteria.

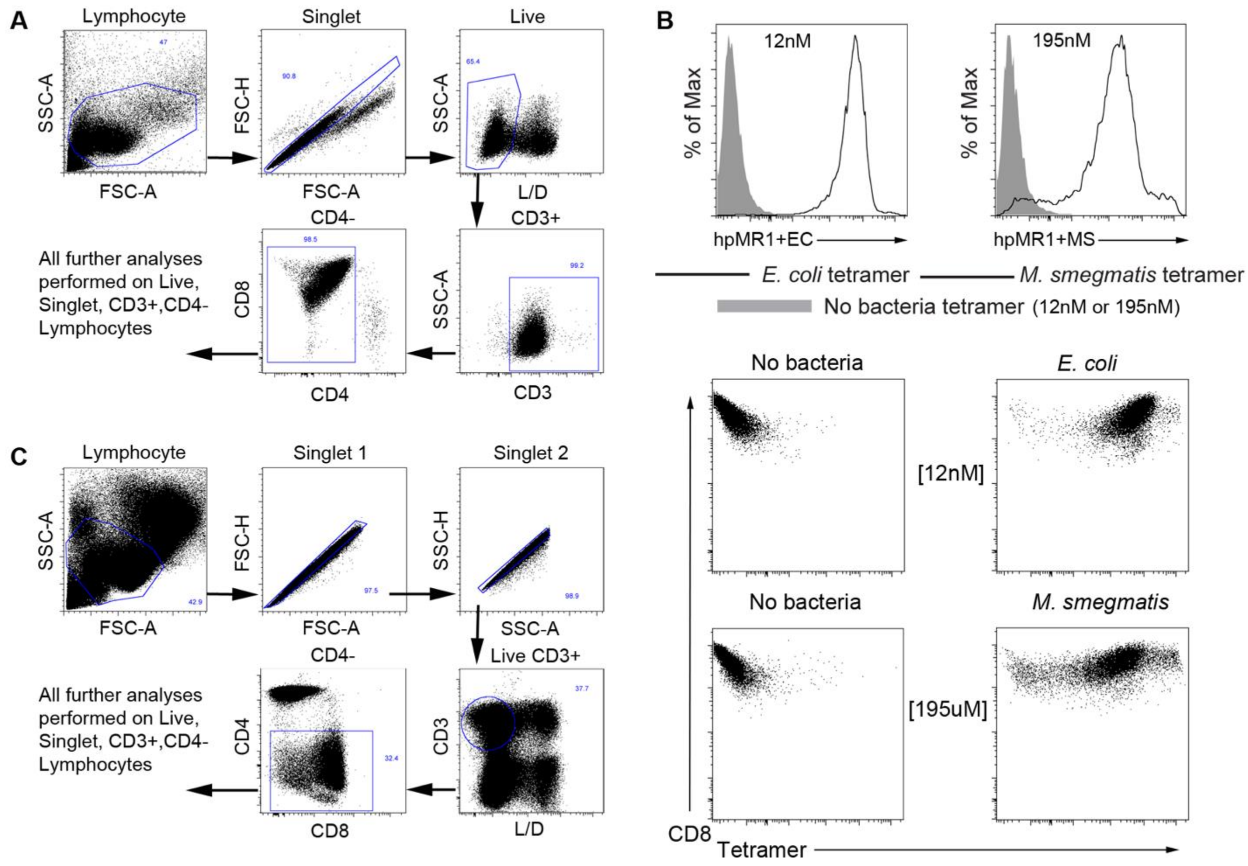

Fig. S2. hpMR1 tetramer staining of MAIT cell clones. A) Gating strategy for MR1T cell clones. The tetramer^{+} parent population consisted of live, singlet, CD3^{+}, CD4^{-} lymphocytes. B) The D426 G11 MR1T cell clone was stained with the hpMR1^{-bac} and $^{+EC}$ tetramers at 12nM per test or the hpMR1^{-bac} and $^{+MS}$ tetramers at 195nM per test. The solid grey histograms represent the hpMR1^{-bac} staining at either concentration. Dot plots for the histogram are shown below. C) Gating strategy for PBMCs. The tetramer^{+} and/or TRAV1-2^{+}CD26^{+}CD161^{+} parent population consisted of live, singlet, CD3^{+}, CD4^{-} lymphocytes.

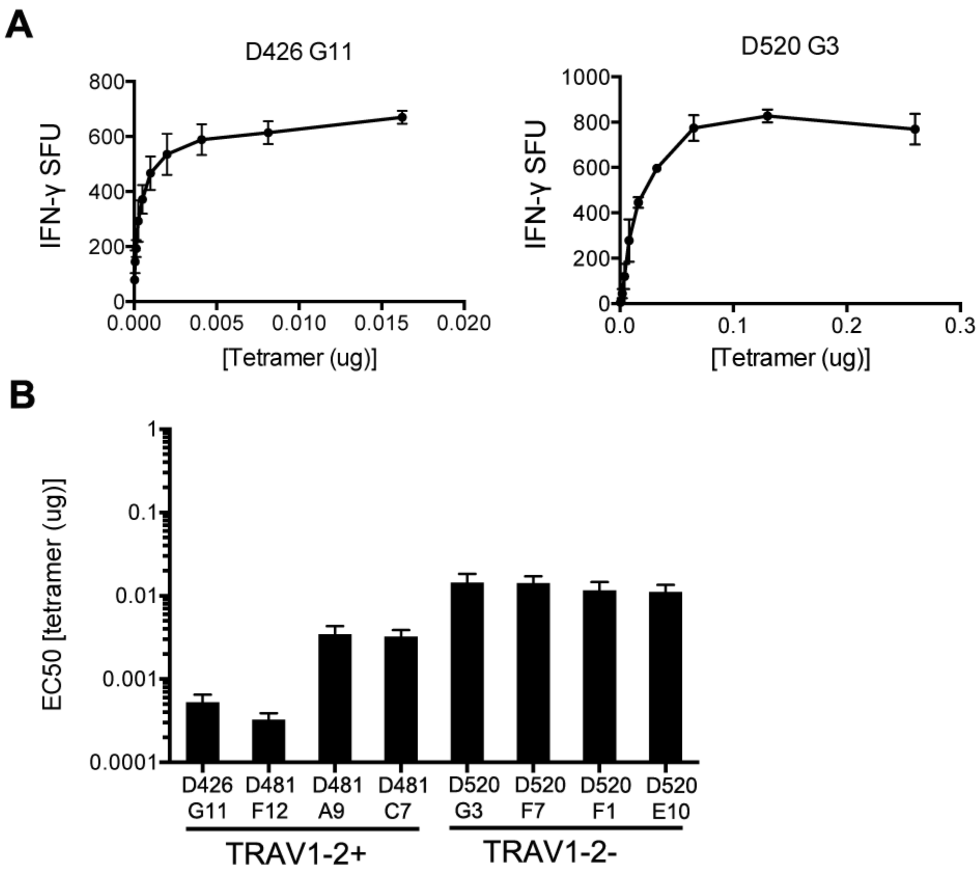

Fig. S3. MR1T clone responses to 5-OP-RU in tetraSPOT assay with MR1/5-OP-RU. A) IFN-γ ELISPOT plates were coated as described with the NIH MR1/5-OP-RU tetramer and MR1T cell clone responses were measured. Shown are representative examples. B) The EC50 was calculated and graphed for each clone.

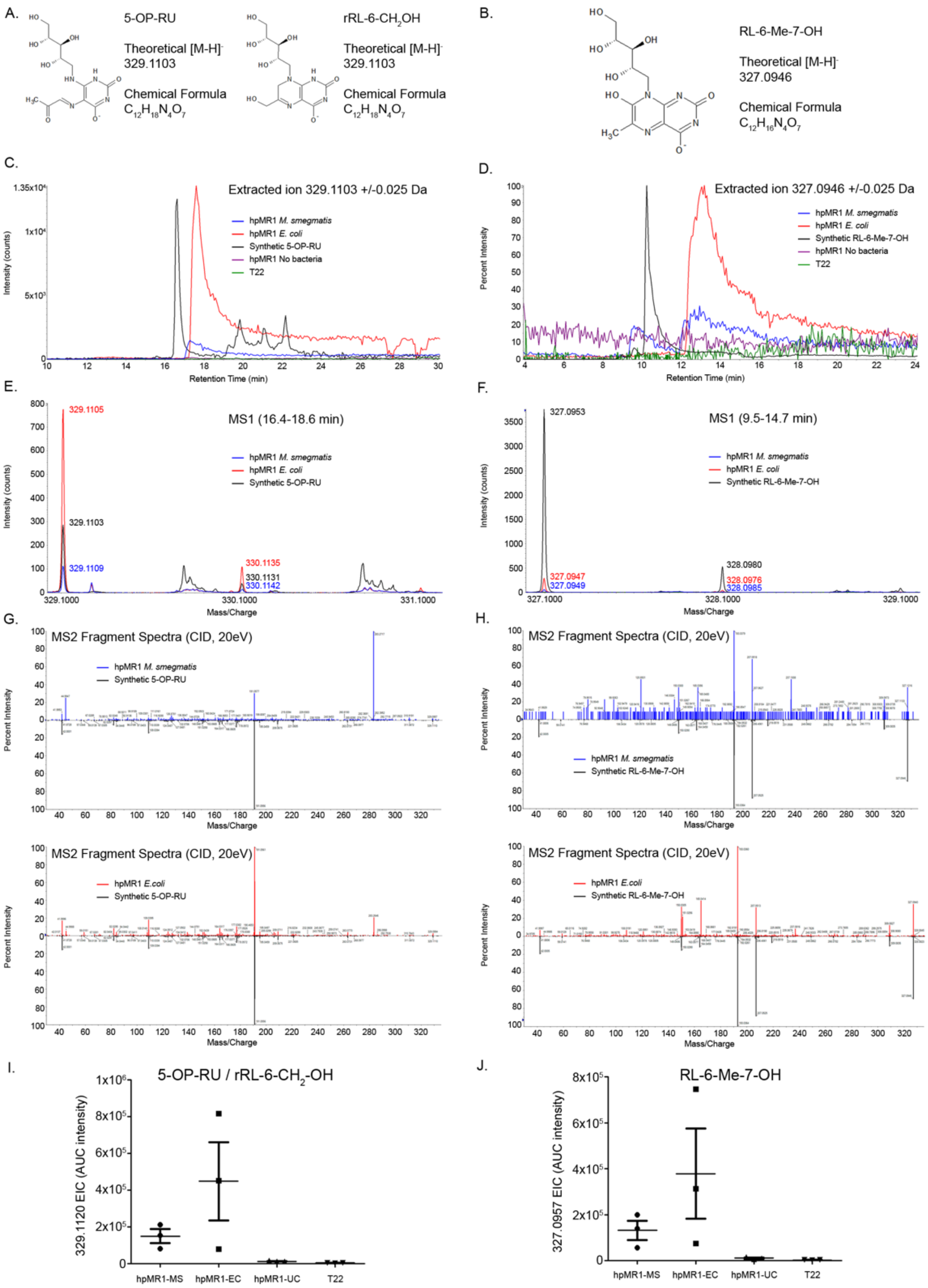

A.
5-OP-RU
Theoretical [M-H]-
329.1103
Chemical Formula
C12H18N4O7
rRL-6-CH2OH
Theoretical [M-H]-
329.1103
Chemical Formula
C12H18N4O7
B.
RL-6-Me-7-OH
Theoretical [M-H]-
327.0946
Chemical Formula
C12H16N4O7
C.
Extracted ion 329.1103 +/-0.025 Da
hpMR1 M. smegmatis
hpMR1 E. coli
Synthetic 5-OP-RU
hpMR1 No bacteria
T22
Intensity (counts)
Retention Time (min)
D.
Extracted ion 327.0946 +/-0.025 Da
hpMR1 M. smegmatis
hpMR1 E. coli
Synthetic RL-6-Me-7-OH
hpMR1 No bacteria
T22
Percent Intensity
Retention Time (min)
E.
MS1 (16.4-18.6 min)
hpMR1 M. smegmatis
hpMR1 E. coli
Synthetic 5-OP-RU
329.1105
329.1103
329.1109
330.1135
330.1131
330.1142
Intensity (counts)
Mass/Charge
F.
MS1 (9.5-14.7 min)
hpMR1 M. smegmatis
hpMR1 E. coli
Synthetic RL-6-Me-7-OH
327.0953
327.0947
327.0949
328.0980
328.0976
328.0985
Intensity (counts)
Mass/Charge
G.
MS2 Fragment Spectra (CID, 20eV)
hpMR1 M. smegmatis
Synthetic 5-OP-RU
MS2 Fragment Spectra (CID, 20eV)
hpMR1 E.coli
Synthetic 5-OP-RU
Percent Intensity
Mass/Charge
H.
MS2 Fragment Spectra (CID, 20eV)
hpMR1 M. smegmatis
Synthetic RL-6-Me-7-OH
MS2 Fragment Spectra (CID, 20eV)
hpMR1 E.coli
Synthetic RL-6-Me-7-OH
Percent Intensity
Mass/Charge
I.
5-OP-RU / rRL-6-CH2-OH
329.1120 EIC (AUC intensity)
hpMR1-MS
hpMR1-EC
hpMR1-UC
T22
J.
RL-6-Me-7-OH
327.0957 EIC (AUC intensity)
hpMR1-MS
hpMR1-EC
hpMR1-UC
T22

Fig. S4. Raw LC-MS data for previously identified ligands 5-OP-RU/rRL-6-CH_2OH and RL-6-Me-7-OH. A-B) [M-H]- ion structure, theoretical monoisotopic mass, and chemical formula for indicated ligands. C-D) Extracted ion chromatogram overlay of the theoretical mass of indicated ligands in hpMR1+MS (blue), hpMR1+EC (red), hpMR1-bac (purple), T22 (green), or synthetic 5-OP-RU (black). E-F) MS1 survey spectra overlay of relevant ion over the indicated retention time range for each indicated sample. Observed m/z for the ion and the respective +1 isotope are labeled and color coded to match the sample trace color. G-H) MS2 fragment spectra of precursor ion in indicated sample mirrored with synthetic ligand. I-J) Intensities of previously identified ligands for 5-OP-RU/rRL-6CH2OH (I) and RL-6-Me-7-OH (J). Data originates from triplicate LCMS injections of the same protein preps.

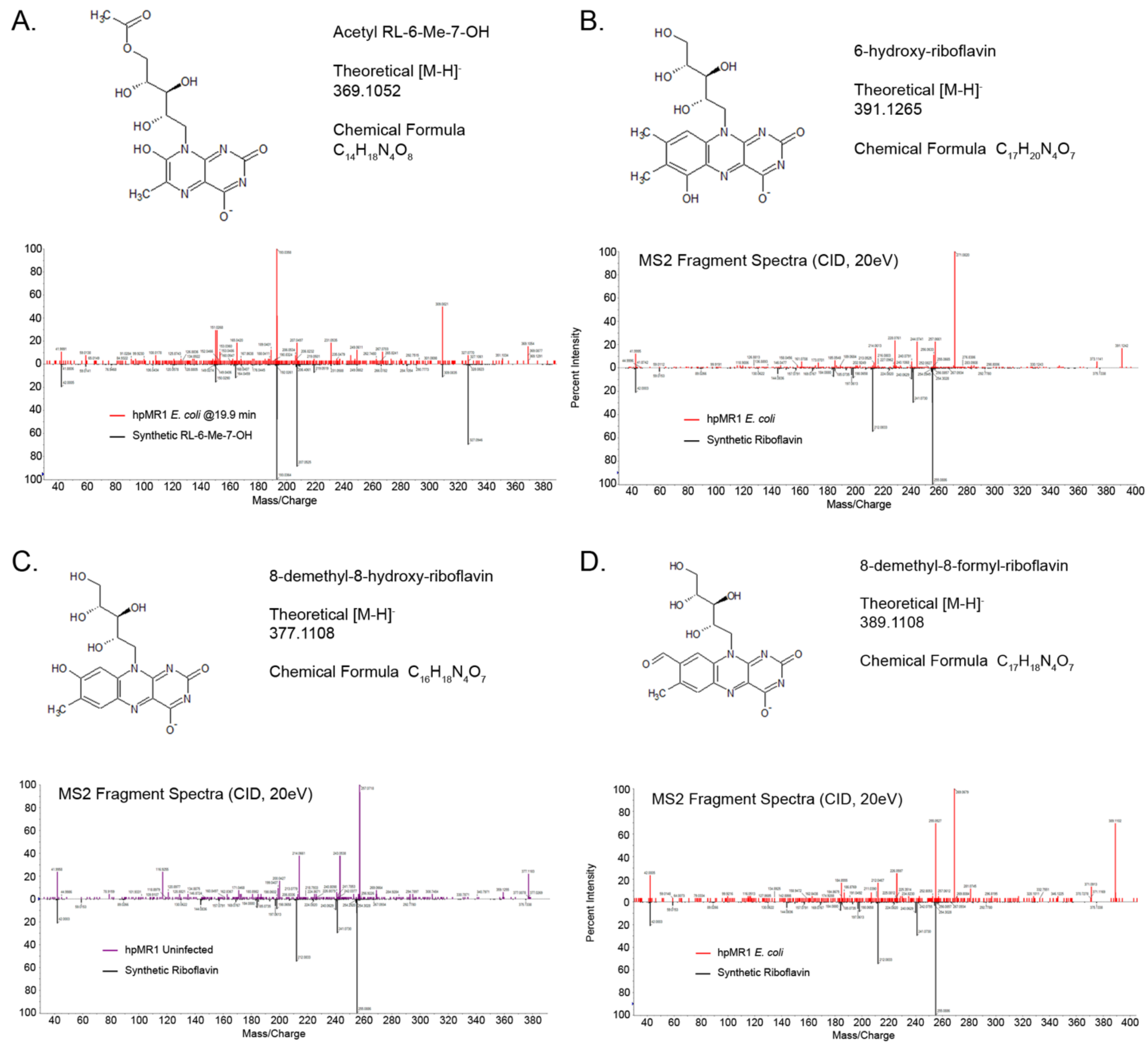

Fig. S5. Raw MS2 fragment spectra for acetyl RL-6-Me-7-OH and riboflavin adducts. Raw MS2 fragment spectra for A) acetyl RL-6-Me-7-OH and the riboflavin adducts B) 6-hydroxy-riboflavin, C) 8-demethyl-8-hydroxy-riboflavin, and D) 8-demethyl-8-formyl-riboflavin. For each molecule, the following data is indicated: [M-H]- ion structure, theoretical monoisotopic mass, and chemical formula for indicated molecule; and MS2 fragment spectra of precursor ion in indicated sample mirrored with the closest synthetic molecule.

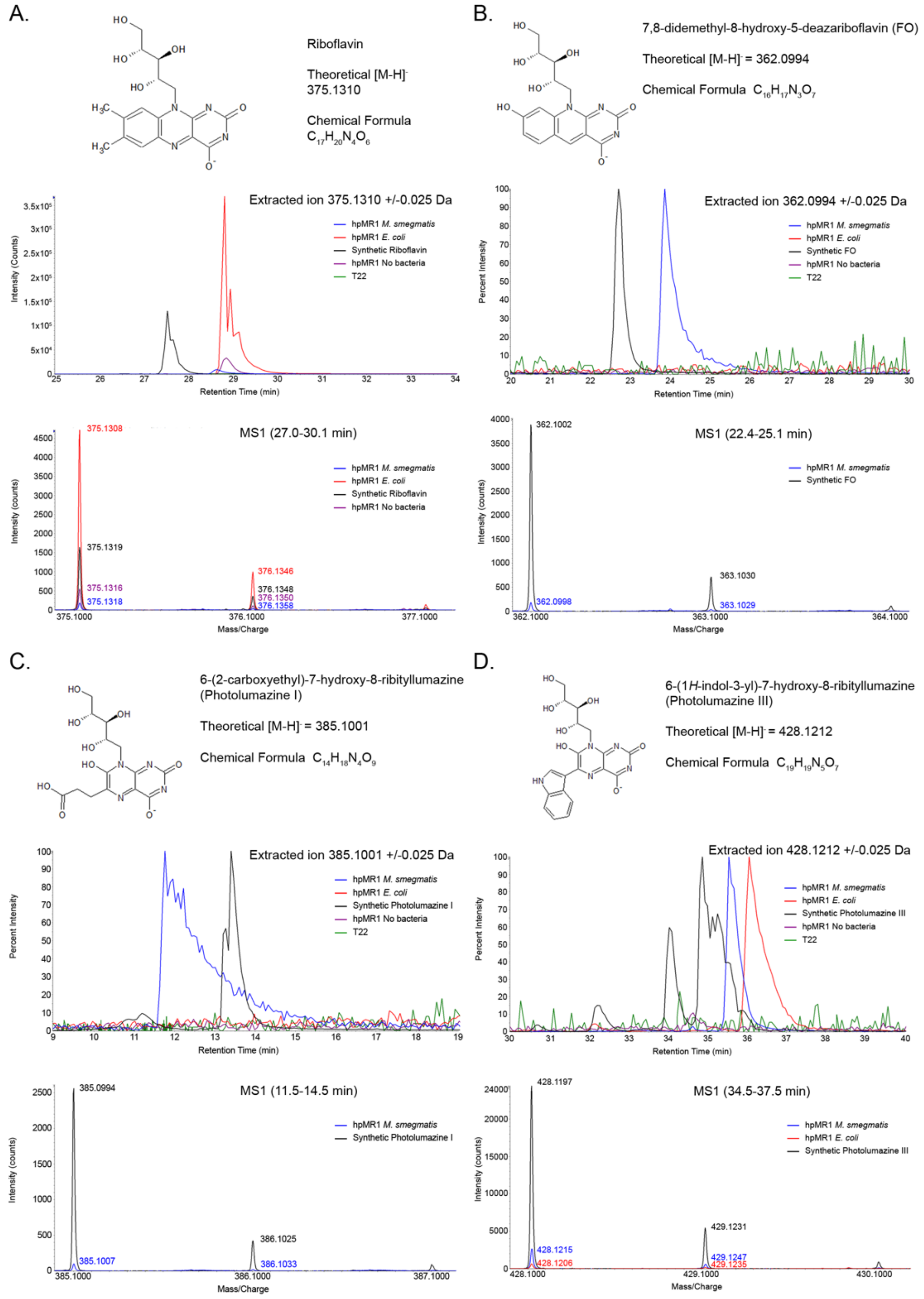

Fig. S6. Raw LC-MS data for newly described MR1 ligands riboflavin, FO, PLI, and PLIII. Raw LCMS data for newly described MR1 ligands A) riboflavin, B) 7,8-didemethyl-8-hydroxy-5-deazariboflavin (FO), C) 6-(2-carboxyethyl)-7-hydroxy-8-ribityllumazine (PLI), and 6-(1*H*-indol-3-yl)-7-hydroxy-8-ribityllumazine (PLIII). For each molecule, the following data is indicated: [M-H]- ion structure, theoretical monoisotopic mass, and chemical formula for indicated molecules; Extracted ion chromatogram overlay of the theoretical mass of indicated molecule in hpMR1+MS (blue), hpMR1+EC (red), hpMR1-bac (purple), T22 (green), or synthetic riboflavin (black); and MS1 survey spectra overlay of relevant ion over the indicated retention time range for each indicated sample. Observed m/z for the ion and the respective +1 isotope are labeled and color coded to match the sample trace color.

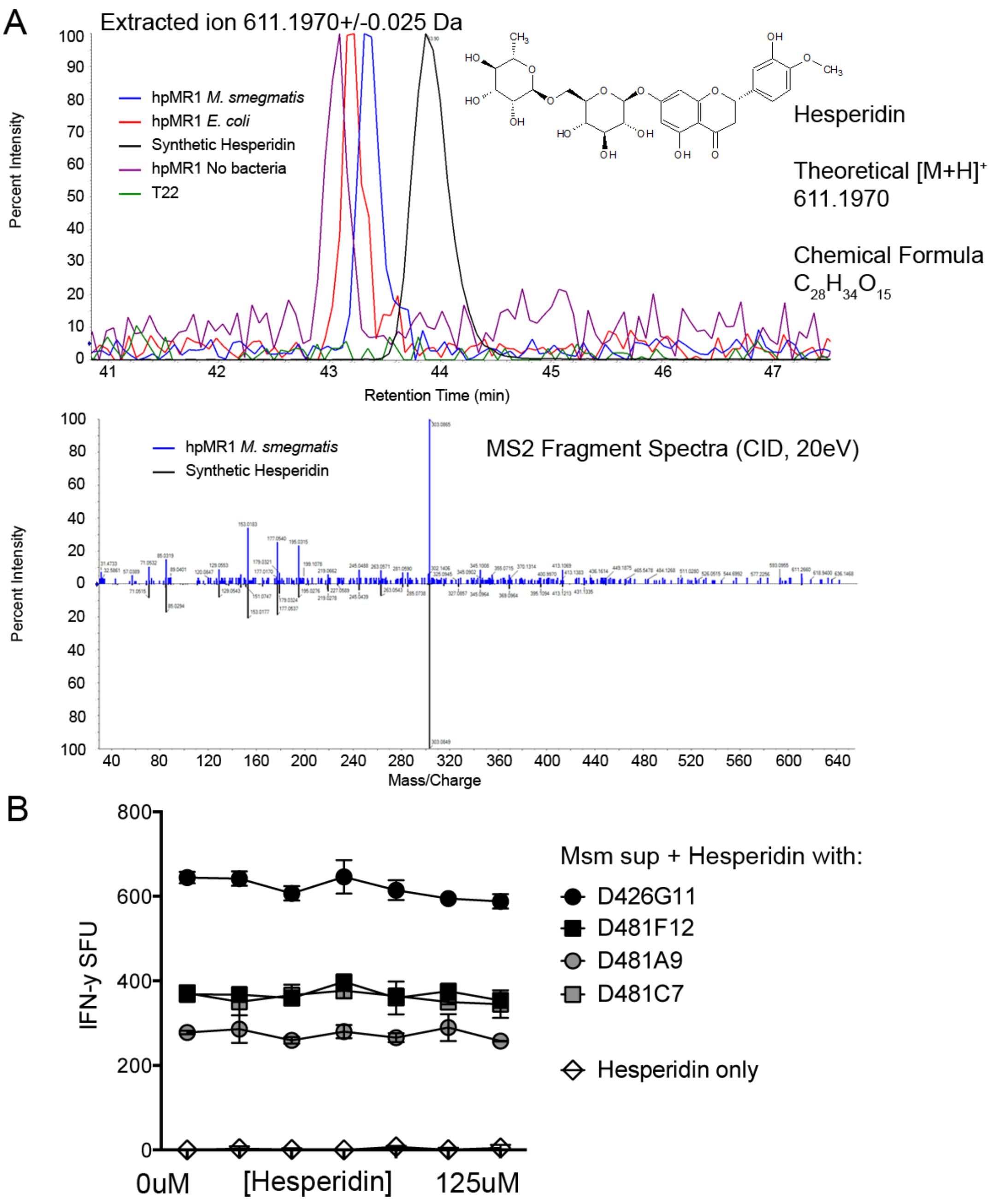

Fig. S7. LC-MS, MS2, and MR1T activation data for hesperidin. A) The following data is indicated: [M-H]- ion structure, theoretical monoisotopic mass, and chemical formula for indicated molecule; and MS2 fragment spectra of precursor ion in indicated sample mirrored with the closest synthetic molecule. B) DC were incubated in an IFN-γ ELISPOT assay with 0-125uM Hesperidin for 1 hour, then incubated or not with a constant volume of supernatant from *M. smegmatis* (Msm sup) for 1 hour. MAIT cells clones were then added overnight and IFN-γ ELISPOTs were enumerated.

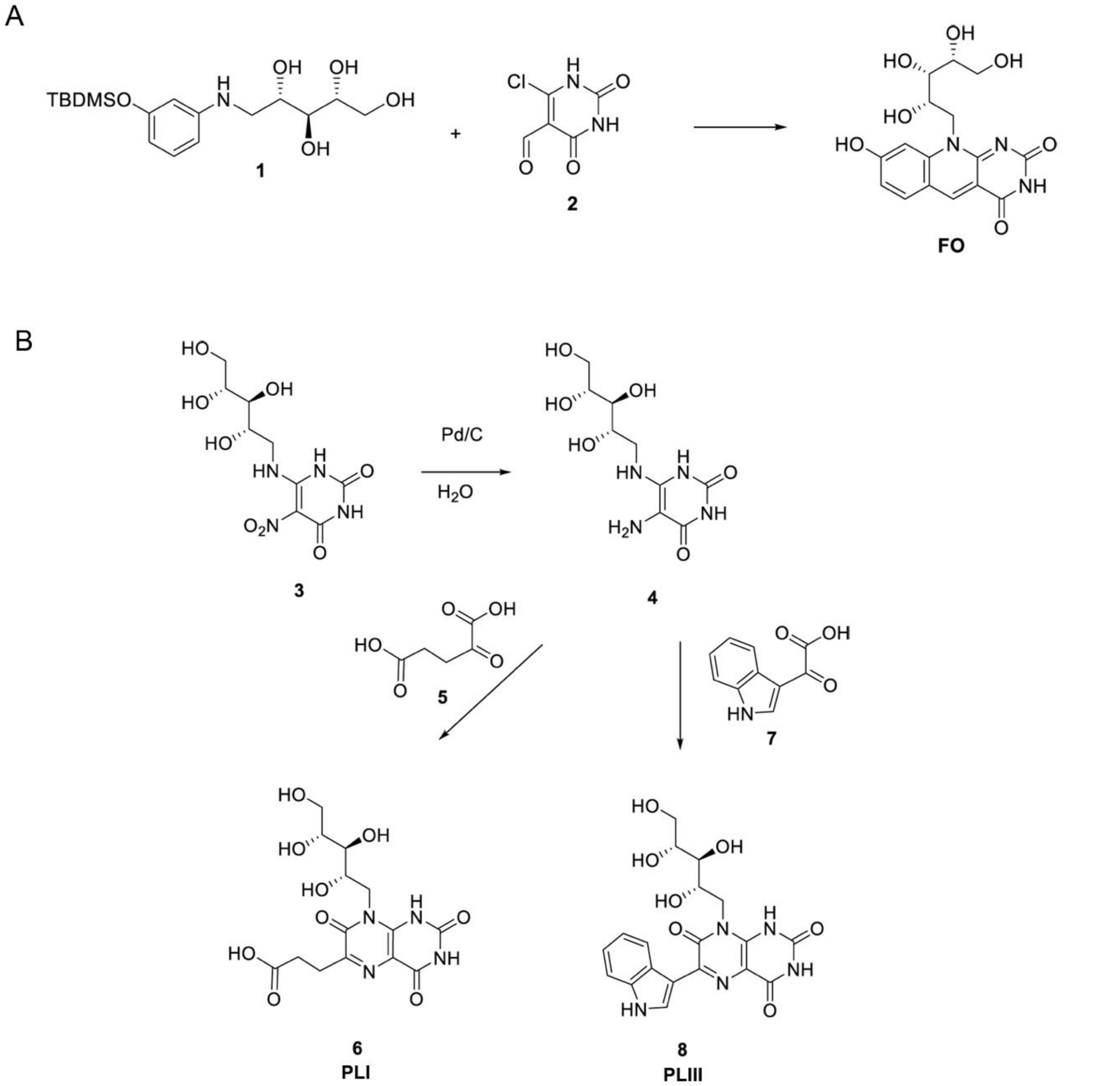

Fig. S8. Chemical synthesis pathway for FO, PLI, and PLIII. Chemical synthesis pathway for A) 7,8-didemethyl-8-hydroxy-5-deazariboflavin (FO). B) 6-(1*H*-indol-3-yl)-7-hydroxy-8-ribityllumazine (PLIII) and 6-(2-carboxyethyl)-7-hydroxy-8-ribityllumazine (PLI). Synthesis details are provided in the Experimental Procedures.
